# Comparison between intra-articular ozone and placebo in the treatment of knee osteoarthritis: A randomized, double-blinded, placebo-controlled study

**DOI:** 10.1371/journal.pone.0179185

**Published:** 2017-07-24

**Authors:** Carlos César Lopes de Jesus, Fânia Cristina dos Santos, Luciana Maria Oliveira Bueno de Jesus, Iara Monteiro, Maria Sonia Sousa Castro Sant’Ana, Virginia Fernandes Moça Trevisani

**Affiliations:** 1 Department of Evidence-Based Medicine, Paulista School of Medicine, Sao Paulo Federal University, Sao Paulo, Sao Paulo, Brazil; 2 Department of Geriatrics and Gerontology, Paulista School of Medicine, Sao Paulo Federal University, Sao Paulo, Sao Paulo, Brazil; 3 ”Dante Pazzanese” Institute of Cardiology, Sao Paulo, Sao Paulo, Brazil; Stavanger University Hospital, NORWAY

## Abstract

**Objective:**

The aim of the trial was to determine the effectiveness of oxygen-ozone injections on knee osteoarthritis concerning pain reduction, joint functional improvement, and quality of life.

**Methods:**

In this randomized, double-blinded, placebo controlled clinical trial, 98 patients with symptomatic knee osteoarthritis (OA) were randomized into two groups receiving intra-articular 20 μg/ml of ozone (OZ) or placebo (PBO) for 8 weeks. The efficacy outcomes for knee OA were the Visual Analogue Scale (VAS), Lequesne Index, Timed Up and Go Test (TUG Test), SF-36, Western Ontario and McMaster Universities Osteoarthritis Index (WOMAC), and Geriatric Pain Measure (GPM).

**Results:**

After 8 weeks of treatment, ozone was more effective than the placebo: VAS [mean difference (MD) = 2.16, p < 0.003 (CI 95% 0.42–3.89)], GPM [MD = 18.94, p < 0.004 (CI 95% 3.43–34.44)], LEQ [MD = 4.05, p < 0.001 (CI 95% 1.10–7.00)], WOMAC (P) [median of diff = 9.999, p = 0.019 (CI 95% 0.000–15.000)], WOMAC (JS) [median of diff = 12.499, p < 0.001 (CI 95% 0.000–12.500)], WOMAC (PF) = [median of diff = 11.760, p = 0.003 (CI 95% 4.409–19.119)], TUG (no statistical difference) and SF-36 (FC) [(MD = -25.82, p < 0.001 (CI 95% 33.65–17.99)], SF-36 (PH) [MD = -40.82, p < 0.001 (CI 95% -54.48–27.17)], SF-36 (GSH) [MD = -3.38, p < 0.001 (CI 95% -4.83–1.93)], SF-36 (SA) [MD = 2.17, p < 0.001 (CI 95% -19.67–8.24), SF-36 (EA) [MD = -35.37, p < 0.001 (CI 95% -48.86–21.89)]. Adverse events occurred in 3 patients (2 in the placebo group and 1 in the ozone group) and included only puncture accidents.

**Conclusions:**

The study confirms the efficacy of ozone concerning pain relief, functional improvement, and quality of life in patients with knee osteoarthritis.

**Trial registration:**

International Standard Randomized Controlled Trial Number Register ISRCTNR55861167

## Introduction

Osteoarthritis (OA) is a group of common, age-related clinical conditions affecting synovial joints [1]. Pathological changes seen in osteoarthritic joints include degradation of the articular cartilage, thickening of the subchondral bone, formation of osteophytes, inflammation of the synovium, and degeneration of ligaments [2]. Typical clinical symptoms are pain and stiffness, particularly after prolonged activity [3]. Articular cartilage is devoid of blood vessels, lymphatics, and nerves, having a limited capacity for intrinsic healing and repair [4]. Symptomatic knee OA is a leading cause of disability, afflicting more than 9.3 million US adults [5]. The diagnosis of osteoarthritis is based on the history and physical conditions, however radiographic findings, including asymmetric joint space narrowing, subchondral sclerosis, osteophyte formation, subluxation and distribution patterns of osteoarthritic alterations can be helpful when the diagnosis is in question [6, 7]. There are no currently approved OA treatments capable of slowing OA-related structural progression or delaying the need for total knee replacement [8, 9]. Ozone (O3) is a triatomic variety of oxygen, applied to the human organism with therapeutic aims, mainly in chronic diseases that receive little benefit with allopathic medicine, such as rheumatic disease osteoarthritis [10]. Probable mechanisms of the action of ozone are: antalgic, anti-inflammatory, and antioxidant effects—by activating the cellular metabolism, reducing prostaglandin synthesis, making the redox system function properly [by reducing oxidative stress through induction of the synthesis of antioxidant enzymes (superoxide dismutase, glutathione peroxidase, and catalase)] and, in addition, amelioration of the tissue oxygen supply through hemoreologic action, vasodilatation, and angiogenesis stimulation [11, 12, 13]. There are few articles on the use of intra-articular ozone in the treatment of knee osteoarthritis and those that exist are clinical series reports [10, 11, 12, 13, 14, 15, 16, 17, 18, 19, 20]. It is interesting to note that in these clinical series, ozone treatment for knee osteoarthritis resulted in a marked clinical improvement in pain and function. Taking into account these results, a double-blinded, PBO controlled clinical trial was designed to assess the efficacy of O3 in patients with symptomatic knee OA concerning pain reduction and functional improvement.

### Objectives

The primary endpoint was pain reduction and the secondary endpoints were functional and quality of life improvements.

## Materials and methods

### Study design

The study comprised patients from São Paulo (Brazil) who were enrolled between November 2010 and March 2015 in three centers, Geriatrics and Gerontology Discipline Clinic, Paulista School of Medicine—Federal University of São Paulo, Pró-Vida—Center for Total Health Assistance and Santo Amaro University. The study protocol was approved by the Ethical Review Board of Paulista School of Medicine—Federal University of São Paulo on October, 10th 2010 under number CEP 1144/10, registered at the International Standard Randomized Controlled Trial Number Register: ISRCTN55861167 and was conducted according to the principles of the Declaration of Helsinki.

#### Inclusion criteria

Eligible patients were male and female subjects aged between 60 and 85 years, with OA of the knee as defined by criteria of the American College of Rheumatology [21], with pain in the affected knee and a confirmatory knee X-ray diagnosis (Kellgren Lawrence grades II-III) [6].

#### Exclusion criteria

Exclusion criteria were patients aged less than 60 years or more than 85 years, Kellgren Lawrence grades I and IV, mental or neurologic deficit, recent knee trauma or suspicion of another joint affection, uncontrolled systemic diseases, thrombocytopenia, bleeding tendencies, use of anticoagulants or antiaggregants, and recent myocardial infarction or stroke.

#### Groups

Patients were randomized into two groups: ozone group (OZ) and placebo group (PBO). Patients from the OZ group received one intra-articular injection of ozone 20 μg/ml—10 ml [10]. Due to the short half-life of ozone (approximately 45 minutes at 20°C, it was freshly generated in the clinics, using an Ozone & Life O&L 3.0 RM generator (Sao Jose dos Campos—Brazil) connected to a pure oxygen source and used immediately for the patient. Ozone generators use oxygen through high voltage tubes with outputs ranging from 4,000–14,000 and produce an ozone-oxygen mixture with concentration ranges extending to 5% [17]. Placebo group patients received an intra-articular injection of 10 ml of air. Each patient received one injection (OZ or PBO) once a week for 8 consecutive weeks. All patients were treated using the sterile injection technique.

#### Sample size calculation

The sample size calculation was determined to guarantee the statistical power mainly for the two primary endpoints. For these variables, a sample size of 40 evaluable patients provided an 80% power to detect a difference of efficacy of 30% between the groups, with a two-sided alpha level of 0.025 and β = 0,20 based on chi squared test [22]. Therefore, a total of 80 evaluable patients were required to analyze the primary endpoints of the study and approximately 96 patients were predefined to be randomized considering a dropout rate of about 20%.

The assortment was not balanced. That occurred because it was made without a control of the total number of patients in each group. Thus, at the end of the study, the relation between the groups was ozone group 1.75 patient: placebo group 1 patient [23]. Because the clinical trial consisted in comparing a new treatment to a pattern, in such a way of acquiring experience and knowledge about the general profile of this treatment, such influences make it necessary to consider the allocation of more than half the sample in this new treatment, even if it occurs some loss of the statistical efficiency [24].

#### Allocation concealment

Opaque envelopes containing the group to which each participant would belong were sequentially numbered and closed by an individual not involved in the study. The envelopes were opened by a nurse in a sequential manner after each patient’s evaluation.

#### Allocation masking

The syringes containing the treatments were delivered by the nurse to the main researcher in closed packages marked with the patient’s initials.

#### Patient inclusion in the study

Study participants attended a baseline visit at which the following procedures were performed: medical history, physical examination, analysis of X-ray of the affected knee and application of the following questionnaires and tests: Visual Analogue Scale (VAS) [25], Lequesne Index [26], Timed Up and Go Test (TUG Test) [27], Short-Form Health Survey (SF-36) [28], Western Ontario and McMaster Universities Osteoarthritis Index (WOMAC) [29], and Geriatric Pain Measure (GPM) [30]. Eligible patients were fully informed of the purpose of the study. All patients that fulfilled the inclusion criteria signed the informed consent prior to enrollment in the trial. They were instructed to continue their medical treatment according to their physicians’ orientations.

#### Randomization

**Fig 1:**
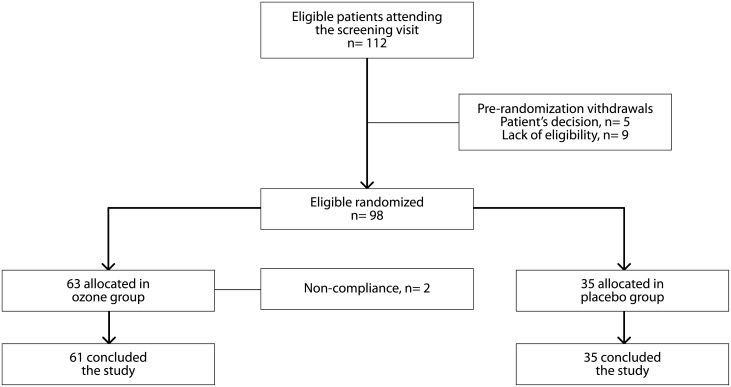
Flow chart of the distribution of study patients.

With the objective of avoiding selection bias, all included participants were sequentially assigned by the researchers to receive OZ or PBO according to a pre-established computer-generated global randomization list. That list was generated by Dr. Fânia Cristina dos Santos (FCS), on November 20th 2010, using software ETCETERA, version 2.46, and constituted 98 numbers with the corresponding treatments (Fig 1). Prior to the beginning of the randomization it was stipulated that group A would be the ozone group and B, the placebo group (Fig 1). Assessments were performed at baseline (visit 1), 4 weeks (visit 2), 8 weeks (visit 3), and 8 weeks after the end of the injections (visit 4). At the follow-up visits, the same procedures as those described for visit 1 were performed.

#### Researcher masking

Patients, the main researcher, and researchers that evaluated the outcomes did not know which group the patients were allocated to.

#### Treatment

Everyone involved in the study was instructed by the main researcher about the way to generate ozone, as well as the cautions to be taken during the process. A member of the study (among the nurses Iara Monteiro and Maria Sonia Sousa Castro Sant’Ana from Paulista School of Medicine—Federal University of Sao Paulo Geriatrics and Gerontology Discipline Clinic and Luciana Maria Oliveira Bueno de Jesus from Pro-Vida—Center for Total Health Assistance LLC and from Santo Amaro University—Medical College) generated the ozone or placebo—according to the criteria of the Randomization Table and in the order of the closed envelopes—and gave the treatment to the main researcher. The nurses were the only members of the study who knew which treatment each patient received. Neither the patient nor the researcher physician had knowledge of whether the syringes dispensed to each patient contained ozone or placebo as they were identical. The substance that was used as placebo was air because it has characteristics identical to those of ozone (except for the smell). Each nurse carefully placed a needle on the beak of the syringe (containing ozone or air) and put a blood collection bottle cover over the needle. In this way, detection of the ozone smell was avoided, as well as its leakage and consequent loss. The syringes containing ozone were maintained in a vertical position, with their beaks upwards, before they received the needle and blood collection bottle cover. Thus, the leakage of ozone from the syringe was avoided since it has a molecular weight higher than oxygen. Next, the syringes were delivered to the main researcher to be used in the treatment.

#### Ozone

The ozone for medical use was obtained from an ozone generator (Ozone & Life—model O&L 3.0 RM, of Brazilian fabrication), composed of a high voltage tube through which medical oxygen (O_2_) passes, dividing into molecules that generate ozone.A 10ml syringe was connected to the exit of the generator and 10ml of the produced gas were collected using the following parameters:

O2 flux=1L/min and Feeder adjusted in position 8

In this way an ozone concentration of 20μg/ml was obtained [10]. During the ozone generation process, the room was kept ventilated to facilitate the process of dispersion of the gas that could escape to the environment.

#### Technique

The knee that was punctured was the more painful and less functional one. Each patient was positioned sitting over a stretcher with the legs bent. The knee to be punctured was submitted to an antisepsis procedure with gauzes profusely soaked in 70° GL alcohol, using round centrifugal movements from the center of the region to be punctured in the direction to its periphery, 3 to 4 times consecutively. The point of entrance of the needle was the femorotibial articular interline, 1.5cm medially to the patellar tendon and 1.5cm bellow the apex of the patella. In cases when the puncture was not possible using this point of entrance, the femorotibial articular interline 1.5cm laterally to the patellar tendon and 1.5cm below the apex of patella was used. The direction in both cases was strictly anteroposterior to avoid the puncture of the Hoffa’s fat pad [31]. In cases when the needle made contact with with the femoral condyle or when there was an error in the knee puncture, another puncture was made. An anesthetic effect was obtained by injecting 0.5ml of 2% lidocaine solution (without vasoconstrictor), with a 1ml syringe and a 30 x 7mm needle, in the route of the puncture [32]. Care was taken to aspirate the syringe before injecting its content to avoid joint effusion that might be present and ensure that the needle was not inside a blood vessel. This was carried out until it was confirmed that the needle was inside the knee joint. Afterwards, the syringe containing ozone or air was connected to the needle used to anaesthetize the route of the puncture and its contents were administered in a slow and continuous manner. The needle and syringe were withdrawn from the knee joint, the region of the puncture was plugged and a dressing was applied with gauze and micropore tape. The patient was instructed not to remove the dressing for at least 30 minutes after the procedure and avoid making efforts with the punctured joint for at least 24 hours. The procedure was carried out once a week, for 8 consecutive weeks.

#### Evaluation tools

**Visual Analogue Scale (VAS)**: VAS is one of the most commonly used instruments to measure pain in the general population as it is considered the most sensitive, reproducible, and simplest pain scale. It is a 10-centimeter line with anchors at both extremities, with the words “without pain” at one end and “unbearable pain” at the other end. The patient is required to mark a point indicating their pain and a 0-100mm ruler is used to quantify the measure [25].

**Lequesne Index**: Lequesne index comprises 10 specific questions for patients with knee osteoarthritis, 5 related to pain or discomfort, 1 to maximum distance walked, and 4 to daily life activities. The score varies between 0 and 24 points, and the higher the score, the worse the pain and function [26].

**Timed Up and Go (TUG test)**: In TUG test, the patient is required to stand up from a chair (height of seat = 45cm and of arms = 65cm), walk 3 meters, return and sit down again, while the time spent performing the test is timed. The proposition of the test is to evaluate balance when sitting, transferring from a sitting position to a standing position, stability when walking and turning when walking without using compensatory strategies. Independent individuals, without balance alterations, perform the test in 10 seconds or less; with independence for basic transfers, they take 20 seconds or less. Individuals who need 30 seconds or more to finish the test are dependent in many daily life activities and moving, presenting a greater risk of falling [27].

**SF-36 Health Survey Instrument**: SF-36 is an instrument of generic evaluation of quality of life, characterized by being easy to apply and understand. The questionnaire contains 36 items, divided into eight aspects: functional capacity, physical aspects, pain, general state of health, vitality, social aspects, and mental health. The score for each item of the questionnaire varies between 0 and 100, where zero is equal to the worst state of health and 100 to the best [28].

**WOMAC (Western Ontario and McMaster Universities) Index**: WOMAC Index contains 24 questions that evaluate pain, stiffness, and physical function during daily life activities (for example, climbing down stairs). The individual is required to indicate the degree of difficulty from 0 (none) to 5 (very strong), of pain and stiffness during the previous 72 hours. The sum of the points given to the 24 items generates a value which varies between 0 and 96; the higher the value, the worse the symptoms of the patient [29].

**Geriatric Pain Measure (GPM)**: GPM was developed to be a multifunctional pain scale, of easy applicability and comprehension to be used in aged populations. It evaluates pain and its impact on mood, daily activities and, predominantly, quality of life. Thus it allows evaluation of the impact of pain on functionality and quality of life in older individuals [30].

#### Statistical analysis

The efficacy analysis was performed in the per-protocol (PP) population defined as all randomized patients who met the inclusion / exclusion criteria, received the treatment and from which data from the baseline, 4th week, 8th week, and 16th week visits were available and who did not present major protocol deviations [33]. Major protocol deviations included lack of fulfillment of the selection criteria, voluntary study exit, and non-compliance with the study treatment. The control population included all randomized subjects who received an air injection. Intention-to-treat analysis (ITT) was not carried out for the 2 patients who dropped out the study because they performed only the baseline evaluation.

#### Data analysis

Data were organized in an Excel spreadsheet for calculation of score variables (Lequesne Index, WOMAC, and Geriatric Pain Measure) and were analyzed using the statistical program SPSS 20.0. Ages were described according to the groups by using the summary measures median, standard deviation and mean and compared between the groups using the Student’s t test. Qualitative characteristics were described according to the groups using absolute and relative frequencies and the existence of an association between the groups was verified using the Chi-squared test, Fisher’s exact test, or likelihood ratio test, and schooling was compared with Mann-Whitney’s test [34]. Percentage alterations of each scale according to the basal values were created. Scales were described according to the groups and evaluation moments using summary-measures, and comparisons between the groups and moments were performed using generalized estimating equations with autoregressive correlation matrices of order 1 between the moments, with normal marginal distribution and identity or logarithmic link function [35]. For models that presented statistical significance, the analysis was followed by Bonferroni’s multiple comparison test to establish between which groups and scales the differences in the scales occurred [36]. Results were illustrated using medium profile graphics, with the respective standard errors and according to the groups, and the tests used a significance level of 5%.

## Results

Of the 112 potential participants, 5 patients decided not to enroll and 9 did not meet the eligibility criteria at the screening visit. Therefore, a total of 98 patients were randomized to the study groups, 63 of which received the ozone treatment (treatment group) and 35 the placebo (control group). However, 2 patients assigned to the treatment group abandoned the study. All patients in the PBO group ended the study.

In spite of the allocation of patients to each group being random, there was a statistical imbalance in marital status between the groups (p = 0.044). In relation to schooling, in the placebo group all patients presented some level of schooling while, in the treatment group, almost 10% of the patients did not have any schooling. The other evaluated characteristics were statistically similar between the groups (p age = 0.533; p sex = 0.489; p race = 0.062; p knee = 0.148) (Table 1).

**Table 1:** Basal characteristics of the study population.

Variable	Group		Total (N = 96)	p
	Placebo (N = 35)	Treatment (N = 61)		
**Age (years)**				0.533**
Mean (SD)	69.5 (7.6)	70.5 (7.2)	70.1 (7.3)	
Median (min; max)	69 (60; 85)	72 (60; 85)	70.5 (60; 85)	
**Schooling**				0.218^£^
None	5 (14.3)	5 (8.2)	10 (10.4)	
Primary school	15 (42.9)	22 (36.1)	37 (38.5)	
1st degree	6 (17.1)	16 (26.2)	22 (22.9)	
2nd degree	9 (25.7)	14 (23)	23 (24)	
Superior	0 (0)	4 (6.6)	4 (4.2)	
**Marital status**				0.044^#^
Single	0 (0)	8 (13.1)	8 (8.3)	
Married	22 (62.9)	31 (50.8)	53 (55.2)	
Separated / divorced	2 (5.7)	5 (8.2)	7 (7.3)	
Widow	11 (31.4)	17 (27.9)	28 (29.2)	
**Sex**				0.489*
Female	30 (85.7)	56 (91.8)	86 (89.6)	
Male	5 (14.3)	5 (8.2)	10 (10.4)	
**Race**				0.062^#^
Caucasian	22 (62.9)	49 (80.3)	71 (74)	
Grayish-brown	12 (34.3)	8 (13.1)	20 (20.8)	
Black	1 (2.9)	2 (3.3)	3 (3.1)	
Asian	0 (0)	2 (3.3)	2 (2.1)	
**Knee**				148
Right	22 (62.9)	29 (47.5)	51 (53.1)	
Left	13 (37.1)	32 (52.5)	45 (46.9)	
**Duration of clinical disease**				
0–5 years	17	25		
6–10 years	12	21		
11–15 years	4	13		
> 16 years	2	2		
**Medication**				
Glucosamine	7	16		
NSAID’s	21	44		
Diacerein	2	4		
Prednisone	2	0		
Paracetamol	2	7		
Dypirone	6	6		
Cat’s claw	0	2		
Chloroquine	1	1		
Opiate (Tramadol^™^)	0	1		
Opiate (Morphine)	0	1		
Opiate (Codeine)	1	1		

Chi-squared test,

*Fisher’s exact test;

^#^Likelihood ratio test;

**t-Student’s test;

^£^Mann-Whitney’s test.

The endpoint pain reduction was evaluated using VAS and GPM. According to the analysis of these tests, the average behavior of the groups over the follow-up was statistically different (p < 0.001). A large decline in the values from the second stage of treatment onwards was observed (p < 0.001). Results were statistically different between the evaluated groups, clearly evidencing pain reduction in patients treated with ozone soon after the beginning of the intervention (p < 0.001) (Table 2).

**Table 2:** Results from VAS and GPM in pain reduction.

Scale	Comparison	Groups		MD	SE	p	Inferior (CI 95%)	Superior (CI 95%)
		Placebo	Treatment					
		Mean (SD) Median (min, max)	Mean (SD) Median (min, max)					
**VAS**	Basal	7.3 (1.8)	7.2 (2.1)	0.06	0.56	<0.999	-1.68	1.79
		8 (4, 10)	7 (2, 10)					
**VAS**	4 weeks	5.1 (2.7)	3.4 (2.6)	1.72	0.56	0.055	-0.02	3.46
		5 (0, 9)	4 (0, 8)					
**VAS**	8 weeks	4.1 (3.1)	1.9 (2.6)	2.16	0.56	0.003	0.42	3.89
		5 (0, 9)	0 (0, 10)					
**VAS**	16 weeks	4.8 (3.6)	1.7 (2.6)	3.16	0.56	<0.001	1.42	4.89
		6 (0, 10)	0 (0, 10)					
**GPM**	Basal	74.4 (16.8)	69.8 (19.8)	4.63	4.96	>0.999	-10.87	20.14
		76.2 (42.8, 97.6)	71.4 (28.6, 100)					
**GPM**	4 weeks	53 (23.2)	34.2 (23.5)	18.75	4.96	0.004	3.25	34.26
		57.1 (4.8, 88.1)	33.3 (0, 80.9)					
**GPM**	8 weeks	41.7 (27.8)	22.7 (23.3)	18.94	4.96	0.004	3.43	34.44
		42.8 (0, 90.4)	16.7 (0, 92.8)					
**GPM**	16 weeks	43.6 (30.3)	20.5 (22.9)	23.10	4.96	<0.001	7.60	38.61
		47.6 (0, 92.8)	14.3 (0, 97.6)					

SD = standard deviation, MD = mean difference, SE = standard error, CI = confidence interval, VAS = Visual Analogue Scale, GPM = Geriatric Pain Measure

Lequesne Index comprises questions related to pain or discomfort and function. It was observed that at the beginning of the study the majority of the individuals were seriously compromised, according to this index. However, at the 8th intervention week, there were a greater proportion of individuals who presented low compromise in the ozone group, compared with the placebo group. In the following evaluation, there was an additional reduction in the index in both groups; however in the ozone group the reduction was more representative and statistically significant when compared to the placebo group (p < 0.001) (Table 3).

**Table 3:** Results from Lequesne Index.

Comparison	Groups		MD	SE	p	Inferior (CI 95%)	Superior (CI 95%)
	Placebo	Treatment					
	Mean (SD) Median (min, max)	Mean (SD) Median (min, max)					
**Basal**	15.9 (3.4)	14.4 (3.7)	1.55	0.95	<0.999	-1.40	4.51
	16 (8.5, 22.5)	13.5 (6.5, 22.5)					
**4 weeks**	12.5 (4.4)	8.6 (4.6)	3.85	0.95	0.001	0.90	6.81
	14 (3.5, 20)	8.5 (0, 18.5)					
**8 weeks**	10.6 (5.1)	6.5 (4.6)	4.05	0.95	0.001	1.10	7.00
	11 (1.5, 19.5)	5.5 (0, 19.5)					
**16 weeks**	10.2 (5.5)	5.8 (4.3)	4.39	0.95	<0.001	1.44	7.35
	10.5 (1, 22)	5 (0, 17)					

SD = standard deviation, MD = mean difference, SE = standard error, CI = confidence interval

Time to perform the activity was measured for each patient at the beginning of the study and during the subsequent moments until the 16th week in TUG test. Results demonstrated a reduction in time (in seconds) for the executed activity in both groups during the treatment. In general, the time reduction was slightly smaller for the ozone group. However, the test was not able to identify a significant difference between the groups.

In relation to pain intensity we observed that the results of WOMAC (pain) demonstrated a reduction in both groups from baseline to the other follow-up moments, however the treated group presented a lower score than the placebo group (p< 0.001). According to WOMAC, the parameter joint stiffness also presented a significant difference in the 8th week of evaluation with better results for the group treated with ozone (p< 0.001). In relation to the parameter physical activities, the ozone group showed better results from the 4th week (p< 0.001). These results remained after the treatment was finished and in the 16th week of follow-up (Table 4).

**Table 4:** Results from WOMAC.

Variable	Time	Groups Median (min, max)		Median of Differences	CI 95% Median of differences		p value
		Placebo	Treatment		Lower	Upper	
**Pain**	**Basal**	50.0 (40, 70)	60.0 (42, 70)	0.000	-9.999	10.000	0.752
	**4 weeks**	20.0 (7, 37)	45.0 (25, 60)	15.000	5.000	25.000	<0.001
	**8 weeks**	10.0 (0, 30)	20.0 (10, 40)	9.999	0.000	15.000	0.019
	**16 weeks**	10.0 (0, 20)	25.0 (2, 52)	14.999	0.000	25.000	0.005
**Stiffness**	**Basal**	37.5 (25, 62)	37.5 (25, 62)	0.000	-12.499	12.499	0.5695
	**4 weeks**	0.0 (0.0, 12)	12.5 (0, 25)	0.000	0.000	12.500	0.0336
	**8 weeks**	0.0 (0.0, 12.5)	12.5 (0, 25)	12.499	0.000	12.500	<0.001
	**16 weeks**	0.0 (0, 0)	0.0 (0, 12)	0.000	0.000	0.000	0.1135
**Functional deficit**	**Basal**	44.1 (26, 68)	50.0 (40, 61)	5.879	-44.100	147.100	0.2973
	**4 weeks**	17.6 (9, 31)	33.8 (26, 51)	16.170	7.350	23.529	<0.001
	**8 weeks**	11.7 (3, 26)	27.9 (14, 35)	11.760	4.409	19.119	0.003
	**16 weeks**	11.8 (2, 24)	25.0 (7, 35)	7.350	1.469	16.180	0.016

Mann-Whitney's test, CI = confidence interval

Quality of life was evaluated using SF-36 Health Survey Instrument. We evaluated domain scores for functional capacity, pain, limitation for physical aspects and limitation for emotional aspects. In all domains there was a medium increase from the baseline score to the other evaluated moments, independent of the group. However, the medium score of the patients was statistically greater in the treated group than in the placebo group, independent of the evaluated moment (p < 0.001). Health status and social aspects showed a statistically significant medium increase between the 4th and the 8th weeks (p < 0.001). Improvement in quality of life was evident for all domains, showing that the ozone group presented better results in this variable (p < 0.001) (Table 5).

**Table 5:** Results from SF-36 Health Survey Instrument.

Variables	Time	Groups		Time	MD	SE	p	Inferior (CI 95%)	Superior (CI 95%)
		Placebo	Treatment						
		Mean (SD) Median (min, max)	Mean (SD) Median (min, max)						
**FC**	**Basal**	27.1 (21.3)	32.4 (23.9)		-12.99	4.64	0.005	-22.08	-3.90
		25 (0, 75)	30 (0,80)						
	**4 weeks**	43.3 (29.3)	58.1 (24)	Basal— 4 weeks	-20.51	2.31	<0.001	-26.59	-14.43
		35 (0, 100)	60 (0, 95)						
	**8 weeks**	49 (27.9)	62.8 (28.8)	Basal— 8 weeks	-25.82	2.97	<0.001	-33.65	-17.99
		45 (0, 100)	70 (0, 100)						
	**16 weeks**	47.1 (28.2)	69.8 (24.7)	Basal— 16 weeks	-27.70	3.42	<0.001	-36.73	-18.67
		45 (0, 95)	70 (15, 100)						
**PH**	**Basal**	27.9 (37.3)	43.4 (43.3)		-23.30	5.62	<0.001	-34.32	-12.27
		0 (0, 100)	25 (0, 100)						
	**4 weeks**	52.1 (43)	80.3 (33.9)	Basal— 4 weeks	-29.93	4.40	<0.001	-41.53	-18.33
		50 (0, 100)	100 (0, 100)						
	**8 weeks**	66.4 (35.8)	86.1 (27.6)	Basal— 8 weeks	-40.82	5.18	<0.001	-54.48	-27.17
		75 (0, 100)	100 (0, 100)						
	**16 weeks**	58.6 (41.5)	89.8 (26.4)	Basal— 16 weeks	-37.72	5.65	<0.001	-52.61	-22.82
		75 (0, 100)	100 (0, 100)						
**GSH**	**Basal**	49.2 (4.6)	50.8 (4.9)		-1.92	0.61	0.002	-3.13	-0.72
		52 (40, 57)	52 (40, 60)						
	**4 weeks**	51.1 (3.6)	52.8 (3.8)	Basal— 4 weeks	-1.96	0.45	<0.001	-3.16	-0.77
		52 (40, 57)	52 (40, 60)						
	**8 weeks**	52.7 (3.5)	54 (3.5)	Basal— 8 weeks	-3.38	0.55	<0.001	-4.83	-1.93
		52 (45, 60)	52 (45, 60)						
	**16 weeks**	50.9 (4.6)	53.9 (4.2)	Basal— 16 weeks	-2.40	0.59	<0.001	-3.95	-0.85
		52 (45, 60)	52 (40, 60)						
**SA**	**Basal**	25.7 (17.7)	29.7 (18.3)		-4.46	2.18	0.041	-8.74	-0.18
		25 (0, 50)	25 (0, 50)						
	**4 weeks**	34.3 (17.2)	39.1 (14.7)	Basal— 4 weeks	-8.99	1.85	<0.001	-13.87	-4.11
		37.5 (0, 50)	50 (13, 50)						
	**8 weeks**	39.6 (16.5)	43.7 (10.9)	Basal— 8 weeks	-13.96	2.17	<0.001	-19.67	-8.42
		50 (0, 50)	50 (13, 50)						
	**16 weeks**	38.6 (17)	43.4 (12)	Basal— 16 weeks	-13.29	2.28	<0.001	-19.32	-7.27
		50 (0, 50)	50 (13, 50)						
**EA**	**Basal**	38.1 (40.5)	53.6 (43.6)		-21.43	5.02	<0.001	-31.27	-11.60
		33.3 (0, 100)	66.7 (0, 100)						
	**4 weeks**	59.1 (42.1)	86.3 (28.8)	Basal— 4 weeks	-26.23	4.45	<0.001	-37.98	-14.48
		66.7 (0, 100)	100 (0, 100)						
	**8 weeks**	72.4 (38.3)	89.6 (24)	Basal— 8 weeks	-35.37	5.11	<0.001	-48.86	-21.89
		100 (0, 100)	100 (0, 100)						
	**16 weeks**	64.8 (41.2)	91.3 (23.5)	Basal— 16 weeks	-31.17	5.45	<0.001	-46.09	-17.32
		100 (0, 100)	100 (0, 100)						

Table 6 shows that according to Bonferroni’s multiple comparison test the questionnaires and tests that presented statistically significant differences between the ozone group (OZ) and placebo group (PBO) were SF-36 (Health Status) (p = 0.030), Lequesne Index (p = 0.001), WOMAC (Pain) (p < 0.001), WOMAC (Physical Capacity) (p = 0.004), VAS (p < 0.001) and Geriatric Pain Measure (GPM) (p < 0.001). Such differences were favorable to the treatment group.

**Table 6:** Multiple comparisons of the percentual changes according to the differences found among groups and evaluation methods.

Variable	Group / Moment	Comparison	Mean Difference	Standard Error	df	p	CI (95%)	
							Inferior	Superior
**Health Status (%)**		4 weeks—8 weeks	-3.09	0.78	1	**<0.001**	-4.95	-1.22
		4 weeks—16 weeks	-0.80	1.13	1	>0.999	-3.49	1.90
		8 weeks—16 weeks	2.29	0.89	1	**0.030**	0.16	4.42
**Social Aspects (%)**		4 weeks—8 weeks	-30.96	9.80	1	**0.005**	-54.42	-7.50
		4 weeks—16 weeks	-28.04	12.76	1	0.084	-58.60	2.52
		8 weeks—16 weeks	2.92	9.70	1	>0.999	-20.31	26.15
**Lequesne (%)**		Placebo—Treatment	-20.49	6.42	1	**0.001**	-33.06	-7.92
		4 weeks—8 weeks	-12.47	2.54	1	**<0.001**	-18.56	-6.38
		4 weeks—16 weeks	-15.68	3.42	1	**<0.001**	-23.86	-7.51
		8 weeks—16 weeks	-3.21	2.49	1	0.590	-9.17	2.74
**WOMAC (Pain) (%)**	**Placebo**	4 weeks—8 weeks	-26.57	6.60	1	**0.001**	-45.94	-7.19
		4 weeks—16 weeks	-14.62	8.39	1	>0.999	-39.24	10.00
		8 weeks—16 weeks	11.94	6.60	1	>0.999	-7.43	31.32
	**Treatment**	4 weeks—8 weeks	-16.00	5.00	1	**0.021**	-30.68	-1.33
		4 weeks—16 weeks	-20.35	6.35	1	**0.020**	-39.00	-1.70
		8 weeks—16 weeks	-4.34	5.00	1	>0.999	-19.02	10.34
	**4 weeks**	Placebo—Treatment	-36.57	9.43	1	**0.002**	-64.25	-8.88
	**8 weeks**	Placebo—Treatment	-26.00	9.43	1	0.088	-53.68	1.68
	**16 weeks**	Placebo—Treatment	-42.29	9.43	1	**<0.001**	-69.97	-14.61
**WOMAC (Stiffness) (%)**		Placebo—Treatment	-21.17	7.97	1	**0.008**	-36.80	-5.55
		4 weeks—8 weeks	-12.63	6.13	1	0.118	-27.29	2.03
		4 weeks—16 weeks	-28.54	7.13	1	**<0.001**	-45.60	-11.48
		8 weeks—16 weeks	-15.91	5.95	1	**0.023**	-30.16	-1.66
**WOMAC (Physical Capacity) (%)**		Placebo—Treatment	-24.09	8.42	1	**0.004**	-40.59	-7.58
		4 weeks—8 weeks	-18.44	4.65	1	**<0.001**	-29.57	-7.32
		4 weeks—16 weeks	-24.15	5.86	1	**<0.001**	-38.18	-10.12
		8 weeks—16 weeks	-5.71	4.07	1	0.483	-15.45	4.04
**VAS (%)**		Placebo—Treatment	-31.84	6.22	1	**<0.001**	-44.03	-19.66
		4 weeks—8 weeks	-17.16	4.73	1	**0.001**	-28.48	-5.84
		4 weeks—16 weeks	-11.69	6.10	1	0.166	-26.28	2.91
		8 weeks—16 weeks	5.47	4.97	1	0.812	-6.42	17.36
**GPM (%)**		Placebo—Treatment	-27.00	6.04	1	**<0.001**	-38.83	-15.16
		4 weeks—8 weeks	-15.71	3.21	1	**<0.001**	-23.39	-8.03
		4 weeks—16 weeks	-15.38	4.22	1	**0.001**	-25.49	-5.27
		8 weeks—16 weeks	0.33	3.19	1	>0.999	-7.32	7.98

Bonferroni’s multiple comparison test

## Safety and tolerability

Intra-articular medical ozone has been shown to be safe for use. Adverse events are rare and comprise acute and transitory pain in the knee at the moment of ozone application [37]. In the present study, adverse effects were collected according to a questionnaire. They were of mild intensity, recorded in 3 patients (2 in the placebo group and 1 in the ozone group) and included only puncture accidents. Treatment compliance was 97.96%, with 2 drop-outs in the ozone group.

## Discussion

Ozone has been used for the treatment of different diseases for over a century. Evidence for the medical use of O3 is, for the most part, based on results of observational studies and case reports in which it has been used in the treatment of several diseases with large effectiveness. This randomized, double-blinded, PBO controlled study presents the results of a 4.5 year clinical trial conducted in patients with knee osteoarthritis receiving intra-articular ozone or a placebo. Although several case reports on the use of intra-articular ozone in the treatment of knee osteoarthritis have been performed [10, 11, 12, 13, 14, 15, 16, 17, 18, 19, 20], none of them was a randomized, double-blinded, PBO controlled, clinical trial. The results of the present study, designed to assess the clinical effects of intra-articular ozone on pain reduction and joint functional improvement, confirm previous positive results obtained with ozone used for the symptomatic treatment of human osteoarthritis [10, 11, 12, 13, 14, 15, 16, 17, 18, 19, 20]. Our results were also corroborated by those of Giombini et al., who used oxygen-ozone for 23 patients with knee osteoarthritis and found it effective in relieving pain and improving function and quality of life [38]. In our clinical trial, ozone elicited a significant reduction in pain intensity and joint function when compared with PBO after 8 weeks of treatment, providing further evidence for its use a treatment for knee osteoarthritis. The effects of ozone increased progressively over time, achieving a maximal effect 8 weeks after the end of the treatment. In agreement with the effects of ozone on pain and joint function, ozone was also able to improve the patient’s health related to quality of life according to SF-36 questionnaire. This is relevant for clinical practice as knee OA presents one of the worst quality of life patterns among musculoskeletal disorders [39]. In spite of the randomization table being generated by a computer program, there were more patients in the ozone group than in the placebo group. There was a difference in basal data in relation to marital status and schooling level, however we do not believe these differences interfered in the observed results. We chose per protocol analysis as the two patients who left the study were not submitted to any intervention. Intra-articular ozone is a safe procedure and complications are the same as for other infiltrations. There is no restriction for the use of ozone in elderly people. The main restrictions for the use of ozone are: acute alcohol intoxication, recent myocardial infarction, hemorrhage from any organ, pregnancy, hyperthyroidism, thrombocytopenia, and ozone allergy [37]. Ozone treatment is considered an adjunctive therapy, especially appropriate for patients with other comorbidities. The action mechanism by which ozone causes analgesia and clinical improvement needs to be further studied as it is not completely clear and we do not have access to the results of the experiments that confirm the effects postulated in the literature. The main limitation of the current study was the lack of imaging exam control to evaluate the impact of the treatment on its evolution. Another limitation was the time for treatment and follow-up. Thus, longer treatment and follow-up periods would confirm the results or elicit more or less favorable results over time. Results of this clinical trial are encouraging and warrant further studies in patients with knee OA to assess the effects of ozone over a longer period of time. The results of the present study suggest that ozone could represent a therapeutic modality for many patients with knee osteoarthritis.

## Conclusions

In conclusion, the weekly administration of 20μg/ml of ozone for 8 weeks reduced osteoarthritis associated pain, improved joint function, and enhanced quality of life of patients with knee osteoarthritis.

## Supporting information

S1 File CONSORT 2010 checklist. (DOC)

S2 File Projeto de pesquisa. (DOC)

S3 File Research project. (DOC)

S4 File Data bank. (XLS)
