# Correction: Comparison between intra-articular ozone and placebo in the treatment of knee osteoarthritis: A randomized, double-blinded, placebo-controlled study

**DOI:** 10.1371/journal.pone.0314494

**Published:** 2024-11-21

**Authors:** Carlos César Lopes de Jesus, Fânia Cristina dos Santos, Luciana Maria Oliveira Bueno de Jesus, Iara Monteiro, Maria Sonia Sousa Castro Sant’Ana, Virginia Fernandes Moça Trevisani

The data in the Placebo and Treatment groups are swapped in Table 4. Please see the correct Table 4 here.

**Table 4:** Results from WOMAC.

Variable	Time	Groups Median (min, max)		Median of Differences	CI 95% Median of differences		p value
		Placebo	Treatment		Lower	Upper	
**Pan**	**Basal**	60.0 (42, 70)	50.0 (40, 70)	0.000	-9.999	10.000	0.752
	**4 weeks**	45.0 (25, 60)	20.0 (7, 37)	15.000	5.000	25.000	<0.001
	**8 weeks**	20.0 (10,40)	10.0 (0, 30)	9.999	0.000	15.000	0.019
	**16 weeks**	25.0 (2,52)	10.0 (0, 20)	14.999	0.000	25.000	0.005
**Stiffness**	**Basal**	37.5 (25, 62)	37.5 (25, 62)	0.000	-12.499	12.499	0.5695
	**4 weeks**	12.5 (0, 25)	0.0 (0.0, 12)	0.000	0.000	12.500	0.0336
	**8 weeks**	12.5 (0, 25)	0.0 (0.0, 12,5)	12.499	0.000	12.500	<0.001
	**16 weeks**	0.0 (0,12)	0.0 (0, 0)	0.000	0.000	0.000	0.1135
**Functional deficit**	**Basal**	50.0 (40, 61)	44.1 (26, 68)	5.879	-44.100	147.100	0.2973
	**4 weeks**	33.8 (26, 51)	17.6 (9, 31)	16.170	7.350	23.529	<0.001
	**8 weeks**	27.9 (14, 35)	11.7 (3, 26)	11.760	4.409	19.119	0.003
	**16 weeks**	25.0 (7, 35	11.8 (2, 24)	7.350	1.469	16.180	0.016
