# Supplementary material for: Comparison between intra-articular ozone and placebo in the treatment of knee osteoarthritis: A randomized, double-blinded, placebo-controlled study
Source: PLoS One. 2017 Jul 24;12(7):e0179185. doi: 10.1371/journal.pone.0179185 (PMC5524330; doi:10.1371/journal.pone.0179185)
Supplement: S2 File — (DOC) [file pone.0179185.s002.doc]

Carlos César Lopes de Jesus
**Comparação entre ozônio intra-articular e placebo no tratamento da osteoartrite do joelho**

**São Paulo**2010

**Ficha catalográfica**

Lopes de Jesus, Carlos César
**Comparação entre o ozônio intra-articular e o placebo no tratamento da osteoartrite do joelho / Carlos César Lopes de Jesus, São Paulo, 2010. 58f.**

Tese (Doutorado) — Universidade Federal de São Paulo. Escola Paulista de Medicina. Programa de Pós-Graduação em Medicina Interna e Terapêutica.
Título em inglês: Comparison between intra-articular ozone and placebo in the treatment of knee osteoarthritis.

1. Osteoartrite; 2. Placebo; 3. Ozônio intra-articular / uso terapêutico; 4. Ensaio clínico randomizado duplo cego; 5. Resultado de tratamento; 6. Efetividade.

Carlos César Lopes de Jesus
**Comparação entre ozônio intra-articular e placebo no tratamento da osteoartrite do joelho**

Projeto de Pesquisa a ser desenvolvido na Escola Paulista de Medicina — Universidade Federal de São Paulo (EPM – UNIFESP) para a obtenção do título de Doutor em Ciências

**São Paulo**2010

Índice

**Lista de abreviaturas, siglas, símbolos e sinais** 06

**Resumo** 07

***Abstract*** 09

**Informações gerais** 11

**Projeto de Pesquisa** 13

**1 Razões e objetivos da pesquisa** 13
1.1 Contexto 13
1.2 Hipótese 17
1.3 Objetivos 17

**2 Plano de Trabalho e Métodos** 18
2.1 Tipo de estudo 18
2.2 Locais 18
2.3 Amostra 18
2.4 Condução do estudo 18
2.5 Seleção 18
2.6 Técnica 19

**3 Variáveis** 21
3.1 Variável primária 21
3.2 Dados complementares 21
3.3 Método estatístico 21
3.4 Resultados 22

**4 Etapas da pesquisa** 23

**5 Cronograma** 24

**6 Relação de materiais necessários** 25

**7 Orçamento** 26

**8 Monitorização da pesquisa** 29

**9 Riscos e benefícios** 30

**10 Propriedades da informação e divulgação da pesquisa** 31

**Bibliografia** 32

**Tabelas** 33 1 Critérios radiológicos 33 2 Critérios diagnósticos da osteoartrite idiopática do joelho 34

**Anexos**1 Modelo do formulário de coleta de dados 35

2 Escala Visual Analógica (EVA) 36

3 Índice Funcional de Lequesne 37 4 Teste Timed Up and Go (TUG) 39
5 SF-36 40
6 WOMAC 47
7 Geriatric Pain Measure (GPM) 50

8 Termo de Consentimento Livre e Esclarecido 52
9 Termo de Retirada do Consentimento Livre e Esclarecido 54

10 Termo de responsabilidade e compromisso do pesquisador
 responsável 55
11 Declaração de condições da instituição 56

12 Currículo Lattes dos pesquisadores 57

**Bibliografia consultada** 58

Lista de abreviaturas, siglas, símbolos e sinais

**EPM-UNIFESP** Escola Paulista de Medicina – Universidade Federal de São Paulo

**LTDA** Sociedade Empresarial de Responsabilidade Limitada

**EVA** Escala Visual Analógica

**TUG** Teste “Timed Up and Go”

**SF-36** Questionário Genérico de Avaliação de Qualidade de Vida - “Medical
 Outcomes Study 36 – Item Short – Form Health Survey

**WOMAC** Western Ontario and McMaster Universities Osteoarthritis Index

**GPM** “Geriatric Pain Measure”

**%** Porcentagem

**α** Erro alfa

**β** Erro beta

**http** Protocolo de Transferência em HiperTexto (do inglês, HyperText
 Transfer Protocol)

**OA** Osteoartrite

**O_3_** Ozônio

**SIDA** Síndrome da Imunodeficiência Adquirida

**µg/ml** Microgramas por mililitro

**cm** Centímetros

**°GL** Graus Gay-Lussac

**ml** Mililitro

**H0** Hipótese de nulidade

**P** % de eventos

**H1** Hipótese alternativa

**A4** Tamanho de papel com 210 mm de largura e 297 mm de altura

**g/cm²** Gramas por centímetro quadrado

**www** Rede Mundial de Computadores (do inglês, World Wide Web)

Resumo

**Título**Comparação entre ozônio intra-articular e placebo no tratamento da osteoartrite do joelho

**Autor**Carlos César Lopes de Jesus

**Instituição**Disciplina de Medicina Interna e Terapêutica

Escola Paulista de Medicina – Universidade Federal de São Paulo (EPM – UNIFESP)

Rua Botucatu, 740 – 3º andar – Vila Clementino

São Paulo, SP - Brazil

CEP: 04023-900

Telefone: +55(11) 5576-4023

Endereço eletrônico: caceloje@gmail.com

Currículo lattes: http://lattes.cnpq.br/5070916128868023

**Contexto**A osteoartrite é a mais comum de todas as doenças articulares, sua importância deriva de seu impacto econômico, em termos tanto de produtividade (principal causa isolada de dias perdidos de trabalho) quanto do custo do tratamento (uso crônico de analgésicos, agentes anti-inflamatórios, condroprotetores e drogas modificadoras da doença). Apesar da etiologia desse distúrbio ainda não ter sido claramente compreendida, constatou-se que a OA é uma família de distúrbios que tem a cartilagem como órgão¬alvo, na qual certos fatores biomecânicos desempenham um papel central e que apresenta alguns fatores de risco (como idade, peso e profissão) também sendo de primordial importância. Como atualmente não existe tratamento capaz de prevenir ou minorar o processo mórbido básico, o tratamento clínico visa principalmente aliviar a dor (1). Desta forma, é de fundamental importância se responder à pergunta da pesquisa: o tratamento da psteoartrite de joelho com ozônio intra-articular é mais efetivo do que o tratamento da osteoartrite do joelho com placebo?

**Objetivo**Determinar se o tratamento da osteoartrite do joelho com ozônio intra-articular é mais efetivo do que o tratamento da osteoartrite do joelho com placebo. A hipótese do pesquisador é que o tratamento da osteoartrite do joelho com ozônio intra-articular seja 30% mais efetivo do que o tratamento com placebo.

**Tipo de estudo**Ensaio clínico multicêntrico, comparativo, randomizado e duplo-cego.

**Locais**Ambulatório da Disciplina de Geriatria e Gerontologia da Escola Paulista de Medicina - Universidade Federal de São Paulo (EPM-UNIFESP), Pró-Vida – Centro de Assistência Integral à Saúde Ltda e Ambulatório da Disciplina de Reumatologia da Faculdade de Medicina da Universidade de Santo Amaro, São Paulo, SP.

**Amostra**Pacientes portadores de osteoartrite do joelho do Ambulatório da Disciplina de Geriatria e Gerontologia da Escola Paulista de Medicina – Universidade Federal de São Paulo (EPM-UNIFESP), da Pró-Vida – Centro de Assistência Integral à Saúde Ltda e do Ambulatório da Disciplina de Reumatologia da Faculdade de Medicina da Universidade de Santo Amaro cujo diagnóstico tenha sido estabelecido de acordo com os critérios do Colégio Americano de Reumatologia.

**Critérios de exclusão**

Serão excluídos os pacientes com idade inferior a 60 anos ou superior a 85 anos, aqueles que não derem seu consentimento para serem incluídos no estudo, pacientes com deficiência mental e / ou neurológica, pacientes com traumas recentes do joelho ou com suspeita de outra lesão articular associada e pacientes que apresentem afecções da articulação coxo-femural.

**Variável**Eficácia do uso intra-articular de ozônio ou placebo no tratamento da osteoartrite de joelho confirmada, clínica e radiologicamente, de acordo com os critérios do Colégio Americano de Reumatologia. A eficácia será mensurada de acordo com: 1) Escala Visual Analógica (EVA); 2) Índice Funcional de Lequesne; 3) Teste “Time Up and Go” (TUG); 4) SF36; 5) Índice de WOMAC (Western Ontario and McMaster Universities Osteoarthritis Index) e 6) “Geriatric Pain Measure”(GPM).

**Método estatístico**O tamanho da amostra foi estimado em 96 indivíduos, considerando-se uma redução da dor no grupo placebo da ordem de 30% e uma diferença significativa de 30% a favor do grupo tratamento. Foi estabelecido um α = 0,05 e um β = 0,20.

**Descritores**1. Osteoartrite; 2. Placebo; 3. Ozônio intra-articular / uso terapêutico; 4. Ensaio clínico randomizado duplo-cego; 5. Resultado do tratamento; 6. Efetividade.

*Abstract*

**Title**Comparison between intraarticular ozone and placebo in the treatment of knee osteoarthritis

**Author**Carlos César Lopes de Jesus

**Institution**Internal Medicine and Therapeutics Discipline

Paulista School of Medicine - São Paulo Federal University (EPM – UNIFESP)

Rua Botucatu, 740 – 3º andar

Vila Clementino - São Paulo, SP

Brazil

Zip Code: 04023-900

Phone: +55(11) 5576-4203

E-mail: caceloje@gmail.com

Lattes curriculum: http://lattes.cnpq.br/5070916128868023

**Context**Osteoarthritis is the most common of all the articular diseases, its importance comes from its economic impact concerning productivity (main cause of lost working days) as well as the cost of treatment (chronic use of analgesics, antiinflammatory agents, condroprotectors and disease-modifying drugs). In spite of the aethiology of such sickness is not yet clearly understood, it has been verified that osteoarthritis is a family of diseases that has the cartilage as the target organ, in which certain biomechanical factors play a central role and that has certain risk factors (as age, wheight and profession) being yet of primordial importance. As nowadays there is no treatment capable of preventing or improving the basic morbid process, clinical treatment aims mainly to relieve pain. This way, it is of vital importance to answer the question: knee arthritis treatment with intraarticular ozone is more effective than the treatment of knee arthritis with placebo?

**Objective**To determine if the treatment of knee osteoarthritis with intraarticular ozone is more effective than the treatment of knee osteoarthritis with placebo. Research hypothesis is that the treatment of knee osteoarthritis with ozone is 30% more effective than the treatment of knee osteoarthritis with placebo.

**Design**Multicenter, comparative, randomized, double-blind clinical trial.

**Setting**Outpatients Department of Geriatrics and Gerontology Discipline of Paulista School of Medicine Federal University of São Paulo, Pró-Vida – Center for Total Health Assistance LLC and Outpatients Department of Rheumatology Discipline - Santo Amaro University – School of Medicine, São Paulo, SP, Brazil.

**Sample**Patients with knee osteoarthritis from Outpatients Department of Geriatrics and Gerontology Discipline of Paulista School of Medicine Federal University of São Paulo (EPM – UNIFESP), Pró-Vida – Center for Total Health Assistance LLC and Outpatients Department of Rheumatology Discipline - Santo Amaro University – School of Medicine.

**Exclusion criteria**

Patients aged less than 60 years or more than 85 years, those who will not give their permission to be included in the study, patients with mental and / or neurologic deficit, patients with recent knee traumas or with suspect of an associated knee lesion and patients with coxofemoral articulation affections.

**Main outcome**Efficacy of intra articular use of ozone or placebo in the treatment of knee osteoarthritis clinically and radiologically according to American College of Rheumatology criteria. Efficacy will be measured in accordance to: 1) Analogic Visual Scale (AVS); 2) Lequesne Functional Index; 3) Timed Up and Go Test (TUG); 4) Medical Outcomes Study 36 – Item Short – Form Health Survey (SF-36); 5) Western Ontario and McMaster Universities Osteoarthritis Index (WOMAC) and Geriatric Pain Measure (GPM).

**Statistical methods**The sample size was estimated in 96 individuals, considering a pain reduction of 30% in placebo group and a significant difference of 30% in favor of the treatment group.

It was established an α = 0,05 and a β = 0,20.

**Headings**1. Osteoarthritis; 2. Placebo; 3. Intra-articular ozone / therapeutical use; 4. Randomized double-blind clinical trial; 5. Treatment result; 6. Effectivness.

Informações gerais

**Locais onde será efetuado o estudo**

1.Ambulatório da Disciplina de Geriatria e Gerontologia da Escola Paulista de Medicina – Universidade Federal de São Paulo (EPM – UNIFESP)
Rua Prof. Francisco de Castro, 105

CEP: 04025-001

São Paulo – SP

Brasil
Telefone: +55 (11) 5575-4848

2. Pró-Vida – Centro de Assistência Integral à Saúde Ltda.
Av. Paes de Barros, 411 – cj. 14 e 15

CEP: 03115-020

São Paulo – SP

Brasil
Telefone: +55 (11) 2307-6202

3. Ambulatório da Disciplina de Reumatologia da Faculdade de Medicina da Universidade de Santo Amaro
Rua Cássio de Campos Nogueira, 2031

CEP: 04829-310

São Paulo – SP

Brasil

**Pesquisador principal**Carlos César Lopes de Jesus, <caceloje@gmail.com>

Mestre em Ciências da Saúde

Doutorando da Disciplina de Medicina Interna e Terapêutica

Escola Paulista de Medicina – Universidade Federal de São Paulo (EPM – UNIFESP)

São Paulo, SP

Telefone: +55(11) 5576-4203

Currículo lattes: http://lattes.cnpq.br/5070916128868023

**Conflito de interesse**Nenhum.

**Fonte de financiamento**Nenhuma.

**Orientadora**Profa. Dra. Virgínia Fernandes Moça Trevisani, < vmoca@uol.com.br >

Professora Doutora Orientadora da Disciplina de Medicina Interna e Terapêutica

Escola Paulista de Medicina – Universidade Federal de São Paulo (EPM – UNIFESP)

São Paulo, SP

Telefone: +55(11) 5575-2970

Currículo lattes: http://lattes.cnpq.br/9054730236021091

**Co-orientadora**Profa. Dra. Fânia Cristina dos Santos, <faniacs@uol.com.br>

Professora Doutora

Chefe da Unidade Ambulatorial da Disciplina de Geriatria e Gerontologia

Escola Paulista de Medicina – Universidade Federal de São Paulo (EPM – UNIFESP) Coordenadora do Grupo de Dor da Disciplina de Geriatria e Gerontologia

Escola Paulista de Medicina – Universidade Federal de São Paulo (EPM – UNIFESP) Coordenadora do Comitê de Dor no Idoso da Sociedade Brasileira para o Estudo da Dor

São Paulo, SP

Telefone: +55(11) 5575-4848

Currículo lattes: http://lattes.cnpq.br/9874664960025710

**Título da pesquisa**Comparação entre ozônio intra-articular e placebo no tratamento da osteoartrite do joelho

**Objetivo**Determinar se o tratamento da osteoartrite do joelho com ozônio intra-articular é mais efetivo do que o tratamento com placebo no controle da dor e na melhora da função articular.

**Custo estimado**R$ 6.079,80

Projeto de Pesquisa

1 Razões e objetivos da pesquisa **1.1 Contexto**A osteoartrite (OA) é um distúrbio das articulações diartrodiais caracterizado clinicamente por dor e limitação funcional, radiograficamente por osteófitos e estreitamento do espaço articular e histopatologicamente por alterações na integridade da cartilagem. Sendo a mais comum de todas as doenças articulares, sua importância deriva do seu impacto econômico, em termos tanto de produtividade (principal causa isolada de dias perdidos de trabalho) quanto do custo do tratamento (uso crônico de analgésicos, agentes anti-inflamatórios, condroprotetores e drogas modificadoras da doença). Apesar da etiologia desse distúrbio ainda não ter sido claramente compreendida, constatou-¬se que a OA é distúrbio cujo órgão-alvo é a cartilagem, no qual certos fatores biomecânicos desempenham um papel central e com fatores de risco (como idade, peso e profissão) de primordial importância. Como atualmente não existe tratamento capaz de prevenir ou minorar o processo mórbido básico, o tratamento clínico visa principalmente aliviar a dor, com as intervenções ortopédicas ficando reservadas para as situações que não podem ser controladas com uma terapia mais conservadora (1).

**Ozônio**O cheiro marcante do ozônio foi registrado pela primeira vez em 1.785 por Van Mauran, mas esse gás só seria "descoberto" em 1.840 pelo químico alemão Christian Frederick Schönbein, na Universidade de Basel, na Suíça. Ele decidiu lhe dar o nome de ozônio (do grego, cheiro) devido ao forte odor da substância (2, 3).

O ozônio é uma forma do oxigênio que ocorre naturalmente na atmosfera. É criado na natureza através da ação dos raios ultravioleta sobre o oxigênio, é formado pela ação de descargas elétricas no oxigênio e também é produzido comercialmente em geradores que liberam uma descarga elétrica sobre um condensador especial contendo oxigênio (2, 3).

Devido ao fato de que o ozônio é feito de três átomos de oxigênio, é conhecido quimicamente como O3 (2, 3).

**O ozônio na Medicina**Usado principalmente para matar vírus, destruir bactérias e eliminar fungos, o ozônio produz importantes benefícios ao corpo humano, como a oxigenação do sangue, o melhoramento da circulação sanguínea e a facilitação da liberação de oxigênio para os tecidos. É também um importante regulador imunológico. Por essa razão, o número de problemas de saúde que podem responder favoravelmente ao tratamento com ozônio é bastante amplo. Os médicos têm usado o ozônio em áreas como a angiologia, dermatologia (incluindo a alergologia), gastroenterologia, terapia intensiva, ginecologia, neurologia, odontologia, oncologia, ortopedia, proctologia, radiologia, reumatologia, cirurgia (incluindo cirurgia vascular) e urologia (2, 3).

De acordo com a Medical Society for Ozone da Europa e o Centro Nacional de Pesquisa Científica de Cuba, os médicos estão utilizando o ozônio nas seguintes doenças: abscessos, acne, AIDS, alergias (hipersensibilidade), fissuras anais, artrite, artrose, asma, neoplasias, esclerose cerebral, distúrbios circulatórios, cirrose hepática, síndrome do climatério, obstipação, úlcera de córnea, cistite, úlceras de decúbito, diarréia, fístulas, doenças causadas por fungos, furúnculos, gangrena, úlcera gastroduodenal, distúrbios gastrointestinais, giardíase, glaucoma, hepatite, herpes simples e herpes zóster, hipercolesterolemia, colite ulcerativa, micoses, distúrbios nervosos, osteomielite, Mal de Parkinson, poliartrite, síndrome de Raynaud, retinite pigmentosa, artrite reumatóide, cicatrizes (depois de radiação), demência senil, septicemia, sinusite, espondilite, estomatite, síndrome de Sudeck (osteoporose pós-traumática), tromboflebite, úlcera nos membros inferiores, vulvovaginites e ferimentos (2, 3).

**O ozônio na Odontologia**De acordo com o dentista alemão Fritz Kramer, o ozônio apresenta as seguintes aplicações:

- na forma de água ozonizada pode ser usado no tratamento de gengivites, periodontites, aftas ou estomatites;
- na forma de spray para limpar área afetada, desinfetar a mucosa bucal e cavidades, e em cirurgias odontológicas;
- na forma de jato para limpar cavidades dos dentes que serão obturados ou submetidos a tratamento de canal (2, 3).

**Como o tratamento com ozônio é aplicado?**Nos últimos sessenta anos, mais de uma dúzia de métodos foram desenvolvidos para a aplicação do ozônio em terapias médicas. Na maioria dos casos, pequenas quantidades de ozônio são acrescidas ao oxigênio puro (geralmente 0,05 parte de ozônio por 99,95 partes de oxigênio para uso interno e 5 partes de ozônio por 95 partes de oxigênio em aplicações externas). A quantidade exata depende de cada caso, pois uma pequena quantidade de ozônio pode ser ineficiente e uma grande quantidade pode bloquear a função imunológica (2, 3).

O ozônio pode ser administrado por via retal, intra-muscular, através da auto-hemoterapia maior e menor, pelo uso externo de água ozonizada, por meio da administração local do gás (usando-se um invólucro de plástico especial) ou utilizando-se óleo ozonizado nas afecções dermatológicas (2, 3).

**Contra-indicações**Constituem contra-indicações ao uso do ozônio: intoxicação alcoólica aguda, infarto do miocárdio recente, hemorragia de qualquer órgão, gravidez, hipertireoidismo, trombocitopenia e alergia ao ozônio (4).

**Mecanismos de ação**O ozônio possui efeitos anti-inflamatório (por atuar diretamente sobre as prostaglandinas e as peroxidases), analgésico e fluidificante do líquido sinovial, além de ser um anti-séptico e germicida de amplo espectro. Ele ainda modula a resposta biológica, o que se manifesta através de uma tendência à normalização da glicose e de outros metabólitos sanguíneos (2, 3).

Acredita-se que o possível mecanismo de ação do ozônio está ligado à geração de produtos secundários. Concebe-se que os compostos orgânicos formados, como os peróxidos orgânicos ozonídeos, exercem diferentes ações biológicas que conferem ao ozônio um conjunto de propriedades terapêuticas, como a melhora da oxigenação tissular, a modulação imunológica, a modulação da liberação de certos autacóides, o poder germicida e a capacidade de regulação metabólica (2, 3).

Contudo, uma das propriedades mais importantes do ozônio é o seu efeito antioxidante, que exerce mediante a estimulação das enzimas pertencentes ao sistema antioxidante do organismo (5)

**Efeitos colaterais**Nos últimos anos, tanto o papel da concentração do ozônio quanto a duração da exposição foram estudados com base em quatro parâmetros: a extensão da hemólise, a glutationa reduzida no meio intraeritrocítico, a viabilidade das células mononucleares do sangue e a produção de citoquinas. Já se demonstrou anteriormente que o ozônio tem efeitos opostos. O ozônio em altas concentrações, prejudica a resposta imune das células e dos líquidos nos animais e nos indivíduos submetidos à exposição crônica. De fato, quando se utilizam altas concentrações de ozônio (acima de 78μg/ml) e, especialmente, quando o sangue é exposto à insuflação constante de ozônio por períodos maiores do que 30 segundos, há uma intensificação progressiva da hemólise, que chega aos 52%, uma diminuição dos níveis de glutationa reduzida no meio intraeritrocítico (de até 47%), uma redução significativa da viabilidade das células mononucleares do sangue e uma produção errática de citoquinas. Por outro lado, quando o contato do ozônio com o sangue dura uns poucos segundos e a concentração de ozônio é inferior a 78μg/ml de sangue, a hemólise não supera os 6%, os níveis de glutationa reduzida no meio intraeritrocítico caem apenas 8,3%, a viabilidade das células mononucleares do sangue não é alterada e há uma produção significativa de citoquinas. Estes resultados se devem, em primeiro lugar, às importantes propriedades antioxidantes do plasma e, também, ao fato de que todas as células metabolicamente ativas dispõem de mecanismos antioxidantes, na forma de diversos sistemas enzimáticos, como a catalase, o superóxido dismutase e a glutationa redutase. A eficiência da homeostase da glutationa é impressionante: em no máximo 30 minutos após o tratamento com ozônio, os níveis de glutationa reduzida no meio intra-eritrocítico voltam ao normal. Já foi demonstrado que a glicose-6-fosfato desidrogenase e a 6-fosfogliconato desidrogenase são enzimas fundamentais que, ao transformarem a glicose em ribulose-5-fosfato, geram a nicotina adenina fosfato dinucleotídeo, o substrato básico para o ciclo de óxi-redução da glutationa. Considerando-se a enorme área exposta pelos eritrócitos é provável que a ação do ozônio se disperse num número quase infinito de alvos da membrana plasmática, dificilmente atingindo o citoplasma, conforme indica a diminuição desprezível e transitória dos níveis de glutationa intra-eritrocítica reduzida (2, 3).

Parece claro, então, que a potencial toxicidade do ozônio não deve impedir seu uso (desde que sejam utilizadas as concentrações adequadas) pois o sangue tem a capacidade de minimizar a formação de radicais livres e de converter oxidantes em variantes menos tóxicas. Na dose correta, o ozônio, como qualquer outra droga, pode ser mais benéfico do que prejudicial. Além disso, vem sendo repensada a conclusão dogmática de que os oxidantes são sempre prejudiciais. Na verdade, a produção, em níveis baixos de variantes altamente reativas pode desempenhar um papel importante na proliferação celular e na defesa e regulação do sistema imunológico. A atividade do óxido nítrico é um exemplo (2, 3).

Em relação às reações adversas da administração intra-articular do ozônio Rifá e Musa observaram somente reações de baixa complexidade (dor imediata ou tardia e febre), o que nos leva a considerar o método como inócuo. Em seu estudo, o efeito colateral mais frequente foi uma dor intensa com irradiação para a perna ou para os músculos com alguns segundos de duração que desaparecia espontaneamente. Inferiu-se que tal dor poderia se dever a um erro de técnica por injetar-se o gás fora da articulação, no espaço periarticular. Isso geralmente ocorreu em pacientes obesas nas quais o espaço ou a linha articular era difícil de se localizar ou, talvez devido ao panículo adiposo abundante, a agulha não tenha chegado a penetrar na articulação. A dor se relacionava com o aumento de volume pelo gás injetado (5).

**Ozônio e osteoartrite**Em 1990, pesquisadores cubanos estudaram 234 pacientes com queixas de dor e problemas relacionados na coluna lombar e sacral, joelho e outras articulações. Um total de 20 injeções intra-musculares de oxigênio e ozônio foram administradas durante um período de 20 dias; uma injeção diária durante os primeiros 10 dias e outras 10 injeções em dias alternados. Todos os pacientes foram cuidadosamente examinados, diagnosticados e avaliados antes do estudo (2, 3).

Os resultados foram impressionantes: 208 pacientes (89%) relataram um completo desaparecimento da dor; 24 (10%) relataram algum grau de melhora e 2 pacientes (1%) relataram que não houve alterações no seu estado de saúde. Durante o seguimento destes pacientes, os pesquisadores verificaram que a maioria dos pacientes permaneceu livre de sintomas durante 3 a 6 meses, enquanto que alguns não sentiram dor por até 11 meses após o tratamento (2, 3).

Devido à melhoria dos métodos de tratamento, estes resultados foram ainda melhores do que os de um estudo cubano prévio realizado com 122 pacientes com osteoartrite, nos quais 71,8% dos pacientes tratados com ozônio relataram uma completa melhora da dor, enquanto que 21,8% relataram uma melhora (6).

No Centro de Estudos Médico-Cirúrgicos em Cuba, 60 pacientes com artrite (a maioria com comprometimento do joelho) receberam uma injeção intra-articular de ozônio por semana durante um total de 10 semanas. Dos 60 pacientes, somente 4 experimentaram o retorno da dor, enquanto a maioria (93,3%) permaneceram livres de sintomas. Os pesquisadores concluíram que este tratamento fácil de ser administrado e de baixo custo produziu o desaparecimento da dor após as primeiras aplicações de ozônio, assim como reduziu a infamação clínica das articulações e restaurou o movimento articular normal (7).

Assim é relevante se responder à pergunta de pesquisa: o tratamento da osteoartrite do joelho com ozônio intra-articular é mais efetivo do que o tratamento da osteoartrite do joelho com placebo em relação ao controle da dor, à melhora da função e à melhora da qualidade de vida?

**1.2 Hipótese**A hipótese é que o tratamento da osteoartrite do joelho com ozônio intra-articular é mais efetivo do que o tratamento da osteoartrite do joelho com placebo em relação ao controle da dor e à melhora da função articular.

**1.3 Objetivos**Determinar se o tratamento da osteoartrite do joelho com ozônio intra-articular é mais efetivo do que o tratamento da osteoartrite do joelho com placebo em relação ao controle da dor e à melhora da função articular.

2 Plano de Trabalho e Métodos

Este projeto de pesquisa será encaminhado para a avaliação do Comitê de Ética em Pesquisa da Escola Paulista de Medicina - Universidade Federal de São Paulo (EPM-UNIFESP). O ensaio clínico terá início após a aprovação deste Comitê.

**2.1 Tipo de estudo**Ensaio clínico multicêntrico, randomizado e duplo-cego.

**2.2 Locais**Ambulatório da Disciplina de Geriatria e Gerontologia da Escola Paulista de Medicina – Universidade Federal de São Paulo (EPM – UNIFESP), Pró-Vida – Centro de Assistência Integral à Saúde Ltda. e Ambulatório da Disciplina de Reumatologia da Faculdade de Medicina da Universidade de Santo Amaro.

**2.3 Amostra
Critérios de inclusão**Serão incluídos os pacientes portadores de osteoartrite do joelho com idades entre 60 e 85 anos, que fornecerem o seu consentimento para serem incluídos no estudo.

**Critérios de exclusão**Os seguintes pacientes serão excluídos: aqueles com idade inferior a 60 anos ou superior a 85 anos, aqueles que não fornecerem o seu consentimento para serem incluídos no estudo, os pacientes com deficiência mental e/ou neurológica, pacientes com trauma recente na articulação do joelho ou com suspeita de outra lesão articular associada e pacientes que apresentem afecções da articulação coxo-femural.

**Critérios de saída**

- Saída voluntária do estudo;
- Ausência em mais de duas sessões consecutivas de tratamento;
- Apresentação de irregularidade de tratamento.

**2.4 Condução do estudo**Os pacientes e familiares serão informados acerca do estudo, seus riscos e características. Os pacientes somente entrarão no estudo após assinarem o Termo de Consentimento Livre e Esclarecido.

**2.5 Seleção**Serão selecionados pacientes com osteoartrite do joelho com diagnóstico firmado utilizando-se os critérios clínicos e radiológicos do Colégio Americano de Reumatologia. Os pacientes selecionados serão randomizados com base numa tabela de números aleatórios para receberem ozônio ou placebo intra-articular. Um integrante do estudo, que não vai tomar parte na avaliação dos pacientes, terá o papel de gerar o ozônio e fornecer ao médico pesquisador ozônio ou placebo e será o único a ter conhecimento do que cada paciente estará recebendo. Nem o paciente, nem o médico avaliador tomarão conhecimento se as seringas a serem dispensadas conterão ozônio ou placebo pois ambas serão idênticas.

**2.6 Técnica**A injeção intra-articular será feita com o paciente deitado, com os joelhos semi-fletidos. O médico estará com as mãos lavadas e enluvadas. A injeção será feita na bolsa supra-patelar para se evitar a inserção da agulha entre superfícies cartilaginosas justapostas. A extremidade superior da patela será identificada e usada como marco para a injeção. A injeção será feita na parte lateral do joelho pois pode ser alcançada mais facilmente. A agulha será inserida 1 a 3 cm abaixo do aspecto superior da patela e será levemente direcionada cranialmente para penetrar na bolsa supra-patelar. Se não for possível obter-se o líquido sinovial, a agulha será redirecionada entre a superfície superior da patela e o sulco patelar do fêmur. Uma outra alternativa de alcance pode ser considerada em pacientes nos quais a deformidade previne a extensão do joelho. Nestes casos, a aspiração e a infiltração serão conseguidas ao se inserir a agulha numa direção ântero-posterior lateral ou medial para o tendão patelar inferior. A agulha passará pela camada de gordura no espaço articular do joelho, entre o côndilo do fêmur e o platô tibial (8).

Far-se-á antissepsia com álcool a 70ºGL; administrar-se-á 1ml de lidocaína a 2% sem vasoconstritor com uma seringa para insulina na região da injeção para se obter um efeito anestésico; conectar-se-á a seringa à via de saída do ozônio e se aspirarão 10ml, quantidade que será injetada na articulação fazendo-se avançar a agulha (número 20) 2 a 3 cm de acordo com o panículo, de modo ligeiramente oblíquo para trás e para dentro até se ter uma sensação de vácuo a qual possibilitará a penetração fácil do gás sem oferecer resistência; aspirar-se-á primeiramente para se esvaziar alguma derrame que por ventura exista e para se precaver de que não se estará dentro de um vaso sanguíneo (8, 9).

Utilizar-se-á uma concentração de 20 µg/ml; para se conseguir esta concentração no equipamento OZONE & LIFE colocar-se-á o calibrador de oxigênio em 1 ml e o relógio do gerador de ozônio na posição 8.

O procedimento será realizado uma vez por semana, sendo que o número máximo de sessões será de 8.

Durante o interrogatório e o exame físico se obterão dados de interesse como idade, sexo, fatores predisponentes, tempo de evolução, tratamento anterior, mobilidade articular e reações adversas, dentre outros.

Para se avaliar a eficácia do uso intra-articular de ozônio ou placebo no tratamento de osteoartrite de joelho confirmada, clínica e radiologicamente, pelos critérios do Colégio Americano de Reumatologia, utilizar-se-ão os seguintes critérios: 1) Escala Visual Analógica (EVA); 2) Índice Funcional de Lequesne; 3) Teste “Time Up and Go” (TUG); 4) SF36; 5) WOMAC (Western Ontário and McMaster Universities Osteoarthritis Index) and 6) “Geriatric Pain Measure” (GPM).

Os pacientes serão avaliados durante a fase de inclusão, após 4 sessões de tratamento, após 8 sessões e 4 meses após o término do tratamento.

**Amostragem**Será utilizada uma amostra probabilística, a amostra casual simples. Os nomes dos pacientes serão submetidos a um sorteio simples sem reposição, utilizando uma tabela de números aleatórios. Assim, serão selecionados 96 indivíduos. A tabela de números aleatórios será geradas no item “números aleatórios” duma planilha eletrônica (Microsoft Excel para Windows 7, Microsoft Inc., Redmond, WA).

**Consentimento livre e esclarecido**Os indivíduos elegíveis para pesquisa baseado no critério de inclusão serão convidados a participar da pesquisa pelo pesquisador principal. Neste momento, serão apresentadas informações sobre a pesquisa (objetivos, riscos, benefícios, e procedimentos aos quais serão submetidos). Confirmado o desejo de participar voluntariamente da pesquisa, será entregue uma cópia do termo de consentimento livre e esclarecido (anexo 7) para que leia o seu conteúdo, entenda-o e possam-se esclarecer eventuais dúvidas. Somente após a assinatura do termo de consentimento é que se formalizará a participação do indivíduo na pesquisa.

3 Variáveis

**3.1 Variável primária**Eficácia do uso intra-articular de ozônio no tratamento da osteoartrite de joelho.

**3.2 Dados complementares**

- Nome
- Idade
- Profissão
- Peso
- Altura

**3.3 Método estatístico
Cálculo do tamanho da amostra**

O cálculo do tamanho da amostra foi determinado para se garantir o poder estatístico principalmente para os dois desfechos principais. Para estas variáveis, um tamanho amostral de 40 pacientes disponíveis provê um poder de 80% para se detectar uma diferença de eficácia de 30% entre os grupos, com um nível de alfa bicaudal de 0,025 (10). Portanto, foi necessário um total de 80 pacientes disponíveis para se analisar os desfechos primários do estudo e aproximadamente 96 pacientes foram pré-definidos para serem randomizados considerando-se uma taxa de evasão de cerca de 20% (10).

**Análise estatística**Os dados serão coletados num formulário padronizado (anexo 10) e os dados armazenados em uma planilha eletrônica de dados (Microsoft Excel para Windows 7. Redmond, WA, EUA). Na qual, cada linha corresponderá a um formulário de coleta de dados e cada linha aos dados coletados. Duas entradas de dados serão realizadas por diferentes digitadores, de forma independente e cega. As controvérsias serão resolvidas por reunião de consenso.

A análise descritiva será realizada calculando o intervalo de confiança de 95% para cada ponto estimado. Os cálculos serão realizados com o auxílio do aplicativo estatístico SPSS20.0.

**Variáveis que serão analisadas**A porcentagem de melhora da dor, da função articular e da qualidade de vida encontradas na pesquisa serão comparadas à hipótese da pesquisa.

**Hipóteses estatísticas**

- Ho:P = 30% (A melhora da dor e da função do joelho com osteoartrite é igual a 30%).
- H1:P ≠ 30% (A melhora da dor e da função do joelho com osteoartrite é diferente de 30%).

**Testes estatísticos**Será utilizada a análise pelo teste t de Student.

**Valor de alfa**Será utilizado o valor de alfa (α) igual ou maior que 0,05 no teste estatístico para rejeitar a hipótese nula.

**3.4 Resultados**Serão utilizados tabelas e gráficos para a apresentação dos dados e os valores de alfa serão apresentados com até quatro casas decimais.

4 Etapas da pesquisa

**Etapas da pesquisa
Etapa I**: **Projeto de Pesquisa**Plano de trabalho para se verificar se a hipótese pode ser negada ou não e para pormenorizar os procedimentos de execução e divulgação da pesquisa Duração de 24 semanas.

**Etapa II: Identificação e seleção dos estudos**Duração de 20 semanas.

**Etapa III: Coleta de dados**
Obtenção dos dados previstos. Duração de 20 semanas.

**Etapa IV: Armazenamento dos dados**Registro e organização dos dados coletados. Duração de 16 semanas.

**Etapa V: Tabulação dos dados**Tabulação e construção de gráficos. Duração de 12 semanas.

**Etapa VI: Análise dos dados**Tentativa de evidenciar as relações expostas entre os resumos e as variáveis. Duração de 12 semanas.

**Etapa VII: Interpretação dos dados**Procura dar significados mais amplos às respostas, vinculando-as a outros conhecimentos e explicitação dos resultados finais, considerados relevantes. Duração de 12 semanas.

**Etapa VIII: Relatório final e artigo original**Exposição geral de pesquisa e elaboração do artigo original. Duração de 16 semanas.

5 Cronograma

| **Mês|Ano** | **Etapa I** | **Etapa II** | **Etapa III** | **Etapa IV** | **Etapa V** | **Etapa VI** | **Etapa VII** | **Etapa VIII** |
| --- | --- | --- | --- | --- | --- | --- | --- | --- |
| **02|2010** | O |  |  |  |  |  |  |  |
| **03|2010** | O |  |  |  |  |  |  |  |
| **04|2010** | O |  |  |  |  |  |  |  |
| **05|2010** | O |  |  |  |  |  |  |  |
| **06|2010** | O |  |  |  |  |  |  |  |
| **07|2010** | O |  |  |  |  |  |  |  |
| **08|2010** |  | O | O |  |  |  |  |  |
| **09|2010** |  | O | O |  |  |  |  |  |
| **10|2010** |  | O | O |  |  |  |  |  |
| **11|2010** |  | O | O |  |  |  |  |  |
| **12|2010** |  | O | O | O |  |  |  |  |
| **01|2011** |  |  | O | O |  |  |  |  |
| **02|2011** |  |  | O | O |  |  |  |  |
| **03|2011** |  |  | O | O |  |  |  |  |
| **04|2011** |  |  |  |  | O |  |  |  |
| **05|2011** |  |  |  |  | O | O | O | O |
| **06|2011** |  |  |  |  | O | O | O | O |
| **07|2011** |  |  |  |  |  | O | O | O |
| **08|2011** |  |  |  |  |  |  |  | O |

**Legenda:** **O:** Planejado | **X:** Realizado

6 Relação de Materiais Necessários

**Materiais de consumo**

| **Item** | **Material** | **Quantidade** |
| --- | --- | --- |
| **01** | Canetas azuis | 004 |
| **02** | Cópias xerográficas | 250 |
| **03** | Papel sulfite branco, 75g/cm², A4 | 500 |
| **04** | Tinta de impressora padrão | 001 |
| **05** | Tinta de impressora padrão | 001 |
| **06** | Seringas de insulina com agulha | 110 |
| **07** | Lidocaína — frasco ampola com 20ml de solução | 006 |
| **08** | Seringas de 10ml com agulha | 110 |

**Materiais permanentes**

| **Item** | **Material** | **Quantidade** |
| --- | --- | --- |
| **01** | Gerador de ozônio | 001 |
| **02** | Regulador de oxigênio | 001 |
| **03** | Cilindro de oxigênio | 001 |

7 Orçamento

**Materiais de consumo**

**Item** Caneta azul **Quantidade** 04 **Valor unidade** R$ 1,50 **Valor total** R$ 6,00 **Justificativa** Utilização no preenchimento das Fichas de Extração de Dados dos
 artigos identificados

**Item** Cópia xerográfica **Quantidade** 500 **Valor unidade** R$ 0,07 **Valor total** R$ 35,00 **Justificativa** Elaboração dos Consentimentos Esclarecidos e Fichas de Extração de
 Dados

**Item** Papel sulfite A4, 75g/cm² **Quantidade** 500 **Valor unidade** R$ 0,04 **Valor total** R$ 20,00 **Justificativa** Utilização na impressão das Fichas de Extração de Dados, dos Relatórios
 Parciais (do aluno, da orientadora e da co-orientadora) e dos Relatórios
 Finais (do aluno, da orientadora e da co-orientadora).

**Item** Tinta de impressora preta padrão  **Quantidade** 01 **Valor unidade** R$ 92,00 **Valor total** R$ 92,00 **Justificativa** Utilização na impressão das Fichas de Extração de Dados, dos Relatórios
 Parciais (do aluno, da orientadora e da co-orientadora) e dos Relatórios
 Finais (do aluno, da orientadora e da co-orientadora).

**Item** Tinta de impressora colorida padrão  **Quantidade** 01 **Valor unidade** R$ 116,00 **Valor total** R$ 116,00 **Justificativa** Utilização na impressão das Fichas de Extração de Dados, dos Relatórios
 Parciais (do aluno, da orientadora e da co-orientadora) e dos Relatórios
 Finais (do aluno, da orientadora e da co-orientadora).

**Item** Seringas de insulina com agulha **Quantidade** 110 **Valor unidade** R$ 0,60 **Valor total** R$ 66,00 **Justificativa** Utilização no tratamento da osteoartrite do joelho.

**Item** Lidocaína frasco — ampola com 20ml de solução **Quantidade** 06 **Valor unidade** R$ 5,80 **Valor total** R$ 34,80 **Justificativa** Utilização na analgesia para o tratamento da osteoartrite do joelho.

**Item** Seringas de 10ml com agulha **Quantidade** 110 **Valor unidade** R$ 1,00 **Valor total** R$ 110,00 **Justificativa** Utilização no tratamento da osteoartrite do joelho.

**Materiais permanentes**

**Item** Gerador de ozônio  **Quantidade** 01 **Valor unidade** R$ 4.500,00 **Valor total** R$ 4.500,00 **Justificativa** Utilização no tratamento da osteoartrite do joelho.

**Item** Regulador de oxigênio  **Quantidade** 02 **Valor unidade** R$ 500,00 **Valor total** R$ 500,00 **Justificativa** Regular a entrada de oxigênio no gerador de ozônio.

**Item** Cilindro de oxigênio **Quantidade** 01 **Valor unidade** R$ 600,00 **Valor total** R$ 600,00 **Justificativa** Necessário para a geração de ozônio.

**Previsão de ressarcimento de gastos aos sujeitos da pesquisa**Não há despesas pessoais para o participante em qualquer momento do estudo. Também não há compensação financeira relacionada à sua participação.

**Direito de indenização**Em caso de dano pessoal, diretamente causado pelos procedimentos propostos neste estudo (nexo causal comprovado), o participante terá direito a tratamento médico na Instituição, bem como às indenizações legalmente estabelecidas.

8 Monitorização da pesquisa

**Medidas para a proteção ou minimização de quaisquer riscos**Todos os procedimentos a que os participantes da pesquisa serão submetidos (aplicação de ozônio ou ar no joelho) serão detalhados, simulados e, só então, o procedimento será realizado. Tudo isso será realizado para a proteção e minimização de quaisquer riscos. A todo instante, o participante da pesquisa será incentivado a fazer questionamentos sobre o estudo ou sobre o procedimento a que estará sendo submetido.

**Medidas de monitorização da coleta de dados**A qualidade dos dados das fichas de coleta será conferida pela comparação dos dados digitados. A conferência será realizada por dois observadores e as discordâncias serão resolvidas por reunião de consenso. Os dados coletados serão armazenados numa planilha eletrônica (Microsoft Excel 2 para Windows 7, Microsoft Inc., Redmond, WA). A conferência será realizada por dois observadores e as discordâncias serão resolvidas por meio de consenso.

**Medidas de proteção à confidencialidade**A confidencialidade dos participantes da pesquisa será mantida sendo que, em nenhum momento, ou por quaisquer meios, existirá a possibilidade de divulgação pública de resultados que permitam identificar os participantes da pesquisa.

**Critérios para suspender ou encerrar a pesquisa**A pesquisa será suspensa se a taxa de recrutamento dos sujeitos da pesquisa for menor que 5 por semana, durante 10 semanas. O Comitê de Ética em Pesquisa será notificado caso isso ocorra.

A pesquisa será encerrada se o arquivo eletrônico matriz (protegido por senha em diretório oculto) e for danificado ou perdido integralmente, impossibilitando a utilização das informações salvas. O Comitê de Ética em Pesquisa será notificado caso isso ocorra.

9 Riscos e benefícios

**Os riscos do estudo são:**O estudo apresenta somente os riscos inerentes à punção do joelho: infecção articular, fraqueza e indisposição e reações urticariformes devido a compostos de veículos em suspensão. Outras complicações podem estar relacionadas com o anestésico local utilizado nas infiltrações. A infiltração endovenosa acidental de lidocaína pode causar graves reações no sistema nervoso central ou cardiovascular. Os efeitos colaterais no sistema nervoso central incluem tontura, alterações visuais, pronúncia ininteligível e convulsões. Foram descritas reações cardiovasculares como bradicardia, hipertensão e colapso cárdio-respiratório. São comuns as reações vaso-vagais imediatas em pacientes com instabilidade vasomotora (9).

Os desconfortos e riscos decorrentes do uso intra-articular do ozônio são dor imediata ou tardia e febre, sendo considerados de baixa complexidade. O mais frequente é uma dor intensa irradiada para a perna ou para o músculo, com alguns minutos de duração, e que desaparece espontaneamente (5).

**Os benefícios do estudo são:**Melhora da audição e da visão, assim como desaparecimento de parestesias em membros inferiores e da sensação de cansaço. Também podem ocorrer melhora da qualidade de vida, da atividade diária e social, melhora da agilidade e do estado de ânimo (5).

10 Propriedades da informação e divulgação da pesquisa

A propriedade dos resultados gerados por esta pesquisa é do seu autor. Os resultados encontrados ao final da pesquisa serão publicados na forma de um artigo original num periódico indexado, independente da confirmação ou não da hipótese da pesquisa.

As cópias do projeto de pesquisa e dos relatórios parciais e final desta pesquisa serão disponibilizadas na rede mundial de computadores (Internet) no site do Centro Cochrane do Brasil (http://www.centrocochranedobrasil.org.br) e entregues à Disciplina de Medicina Interna e Terapêutica da Escola Paulista de Medicina - Universidade Federal de São Paulo (EPM – UNIFESP) para a obtenção do título de Doutor em Ciências.

Bibliografia

1. Schnitzer TJ. Osteoartrose (Doença óssea Degenerativa). In Bennett, JC; Plum, F. Cecil Tratado de Medicina Interna 20ª edição. Rio de Janeiro: Editora Guanabara Koogan; 1997. p. 1677-1681.
2. Leite RC. Terapias Bioxidativas 1ª edição. Curitiba: Corpo Mente Publicações; 1999. p. 18-43.
3. Leite RC. Ozônio 1ª edição. Curitiba: Corpo Mente Publicações; 1999. p. 5-17.
4. Al-Jazira AA; Mahmoodi SM. Painkilling effect of ozone-oxygen injection on spine and joint osteoarthritis. Saudi Medical Journal 2008; 29(4), 553-557. Dubai. United Arab Emirates.
5. Delgado Rifá Eraclio, Quesada Musa Juan Vicente. Ozonoterapia intraarticular en la enfermedad artrósica de rodilla. Rev Cubana Ortop Traumatol [revista en la Internet]. 2005 Jun [citado 2010 Jul 10] ; 19(1). Disponível em: http://scielo.sld.cu/scielo.php?script=sci_arttext&pid=S0864-215X2005000100005&lng=es.
6. Altman N. The Oxygen Prescription – The Miracle of Oxidative Therapies. Rochester: Healing Arts Press; 2007. p. 162 e 163.
7. Altman N. The Oxygen Prescription – The Miracle of Oxidative Therapies. Rochester: Healing Arts Press; 2007. p. 166.
8. Furtado R, Natour J. Infiltrações no aparelho locomotor – técnicas para realizaçao com e sem o auxílio de imagem. Porto Alegre: Artmed Editora S.A.; 2011.
9. Neustadt DH. Intra-articular injections for osteoarthritis of the knee. Cleveland Clinic Journal of Medicine. 2006.;73(10) 897-911..
10. Nagib H. Metodologia de Estudos em Ciências da Saúde – Como Planejar, Analisar e Apresentar um Trabalho Científico. São Paulo: Roca; 2.004.

Tabelas

**Tabela 1
Critérios radiológicos**

Classicamente o diagnóstico de osteoartrite tem se baseado em critérios radiológicos desde que Kellgren e Lawrence, em 1.957, publicaram seu trabalho descrevendo alterações radiológicas da osteoartrite. A tabela a seguir mostra os aspectos relevantes evidenciados neste estudo para o diagnóstico radiológico da osteoartrite.

| **01** | **Osteófitos nas margens articulares ou no caso dos joelhos, nas espinhas tibiais.** |
| --- | --- |
| **02** | Ossículos periarticulares; particularmente em interfalangeanas distais e proximais. |
| **03** | Estreitamento da cartilagem articular associada com esclerose de osso subcondral. |
| **04** | Pequenas áreas pseudocísticas com paredes escleróticas situadas usualmente no osso subcondral. |
| **05** | Forma alterada das margens ósseas, particularmente na cabeça do fêmur. |

As combinações destas alterações geram uma escala de gravidade de 0 a 4 conforme as alterações acima descritas sejam ausentes (0), duvidosas (1), mínimas (2), moderadas (3), e graves (4). No entanto, algumas limitações evidenciadas no estudo de Kellgren e Lawrence dificultam a avaliação do espaço articular, pois as radiografias de joelhos são feitas sem sustentação de carga.

**Kellgren JH, Lawrence JS. Radiological assessment of osteo arthrosis. Ann Rheum Dis. 1957;16(4):494-501.**

**Tabela 2
Critérios diagnósticos da osteoartrite idiopática do joelho**

| **Clínico e Laboratorial** | **Clínico e Radiológico** | **Clínico** |
| --- | --- | --- |
| **Dor em pelo menos 5 dos 9** | Dor em pelo menos 1 dos 3 | Dor em pelo menos 3 dos 6 |
| **Idade > 50 anos** | Idade > 50 anos | Idade > 50 anos |
| **Rigidez < 30min** | Rigidez < 30min | Rigidez < 30min |
| **Crepitação** | Crepitação | Crepitação |
| **Alargamento ósseo** | + | Alargamento ósseo |
| **Dolorimento ósseo** | Osteófitos | Dolorimento ósseo |
| **Discreto aumento da temperatura** | — | Discreto aumento da temperatura |
| **VHS < 40mm/hora** | — | — |
| **Fator reumatóide < 1:40** | — | — |
| **Fluido sinovial de AO** | — | — |
| **92% sensível** | 91% sensível | 95% sensível |
| **75% específico** | 86% específico | 69% específico |

VHS = Velocidade de Hemossedimentação; FS OA = sinais de OA no fluido sinovial (claro, viscoso ou contagem de leucócitos <2 células/mm3).

Uma alternativa para o diagnóstico clínico seria 4 de 6, que é 84% sensível e 89% específica.

**Altman R, Ash E, Bloch D, Bole G, Borenstein K, Brandt K, et al. Development os criteria for the classification and reporting os osteoarthritis. Classification of osteoarthritis of the knee. Diagnostic and therapeutic Criteria Committee of the American Rheumatism Association. Arthritis Rheum. 1986; 29(8):1039-49.**

Anexos

**Anexo 1
Modelo do formulário para coleta de dados**

1. Identificação

| 0 | 1 | 2 | 3 | 4 | 5 | 6 | 7 | 8 | 9 |
| --- | --- | --- | --- | --- | --- | --- | --- | --- | --- |
| 0 | 1 | 2 | 3 | 4 | 5 | 6 | 7 | 8 | 9 |
| 0 | 1 | 2 | 3 | 4 | 5 | 6 | 7 | 8 | 9 |

**2. Nome do pesquisador**_______________________________________________________________________

**3. Data de preenchimento** ___|___|_____

**4. Hora do preenchimento** ___h___min

**5. Nome do participante da pesquisa**_______________________________________________________________________

**6. Sexo 1** Feminino **2** Masculino

**7. Data de nascimento** ___|___|_____

**8. Profissão**_______________________________________________________________________

**9. Estatura**  _______ m

**10. Peso** _______ kg

**11. Intensidade da dor**_______________________________________________________________________

**12. Sessão de tratamento**_______________________________________________________________________

**Anexo 2**

**Escala Visual Analógica (EVA)**


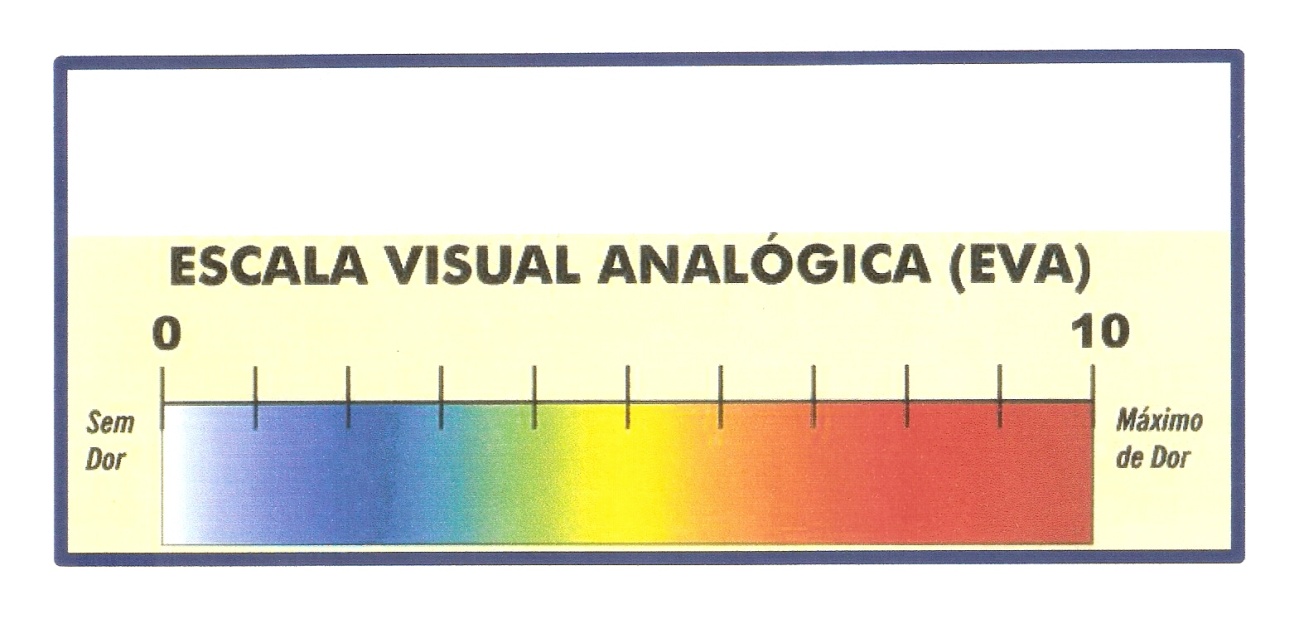


**Price DD. The validation of visual analogue scales as ratio scale measures for chronic and experimental pain. Pain. 1983; 17:45-56.**

**Anexo 3**

**Índice Funcional de Lequesne**

**Dor ou incômodo Pontos**

**Noturno**Não 0 ⬜
Aos movimentos ou em determinada postura 1 ⬜
Imóvel 2 ⬜

**Aos primeiros movimentos matinais**Menos de 1 minuto 0 ⬜
Entre 1 e 15 minutos 1 ⬜
Mais de 15 minutos 2 ⬜

**Ao permanecer em pé ou nas pontas dos pés por meia hora**Não 0 ⬜
Sim 1 ⬜

**Ao caminhar**Não 0 ⬜
Somente após certa distância 1 ⬜
Muito rapidamente e de modo progressivo 2 ⬜

**Ao subir um andar sem ajuda dos braços**Não 0 ⬜
Sim 1 ⬜

**Distância máxima da marcha**Nenhuma limitação 0 ⬜
Limitado, mas superior a 1 hora 1 ⬜
Aproximadamente 1 km (aproximadamente 15 minutos) 2 ⬜
500 a 900 m (aproximadamente 8 a 15 minutos) 3 ⬜
300 a 500 m 4 ⬜
100 a 300 m 5 ⬜
Menos de 100 m 6 ⬜
Com uma muleta + 1 ⬜
Com duas muletas +2 ⬜

**Dificuldade da vida cotidiana**Para subir um andar 0 a 2 _____
Para descer um andar 0 a 2 _____
Para agachar-se completamente 0 a 2 _____
Para caminhar num terreno irregular 0 a 2 _____

**Total**
0 Ausência de dificuldade.
0,5 a 1,5 De acordo com o grau de dificuldade.
2 Impossível.

**Adaptado de: Lequesne MG. The algofunctional indices for hip and knee osteoarthritis. J Rheumatol. 1997; 24: 779-781.**

**Anexo 4
Teste "Timed Up and Go (TUG)"**

No "Timed Up and Go-TUG Test", o paciente é solicitado a levantar-se de uma cadeira (altura do assento de 45cm e dos braços de 65cm), deambular 3 metros, retornar e sentar-se novamente, enquanto o tempo despendido na realização desta tarefa é cronometrado. A proposta do teste é avaliar o equilíbrio sentado, transferências de sentado para a posição em pé, estabilidade na deambulação e mudança do curso da marcha sem utilizar estratégias compensatórias. Indivíduos independentes, sem alterações no equilíbrio, realizam o teste em 10 segundos ou menos; com independência em transferências básicas, gastam 20 segundos ou menos. Já os indivíduos que necessitam de mais de 30 segundos para realizar o teste são dependentes em muitas atividades de vida diária e na mobilidade, apresentando riscos aumentados de cair.

**Podsiadlo D, Richardson S. The Timed “Up & Go”: a test of basic functional mobility for frail elderly persons. Journal of the American Geriatrics Society 1991; 39:142-48.Oliveira DLC, Goretti LC, Pereira LSM.**

**Anexo 5
SF-36 (Questionário Genérico de Avaliação de Qualidade de Vida — “Medical Outcomes Study 36 — Item Short — Form Health Survey)**

**1. Em geral, você diria que a sua saúde é (circule uma alternativa):**
1 Excelente
2 Muito boa
3 Boa
4 Ruim
5 Muito ruim

**2. Comparada a um ano atrás, como você classificaria sua saúde em geral, agora (circule uma alternativa)?**1 Muito melhor agora do que um ano atrás
2 Um pouco melhor agora do que um ano atrás
3 Quase a mesma de um ano atrás
4 Um pouco pior agora do que um ano atrás
5 Muito pior agora do que há um ano atrás

**3. Os seguintes itens são sobre atividades que você poderia fazer atualmente durante um dia comum. Devido à sua saúde, você tem dificuldade para fazer essas atividades? Neste caso, quanto (circule uma alternativa para cada atividade)?**

**Atividades vigorosas, que exigem muito esforço, tais como correr e levantar objetos pesados.**
1 Sim, dificulta muito
2 Sim, dificulta um pouco
3 Não, não dificulta de modo algum.

**Atividades moderadas, tais como mover uma mesa pesada, passar aspirador de pó, jogar bola e varrer.**1 Sim, dificulta muito
2 Sim, dificulta um pouco
3 Não, não dificulta de modo algum.

**Levantar ou carregar mantimentos.**1 Sim, dificulta muito
2 Sim, dificulta um pouco
3 Não, não dificulta de modo algum.

**Subir vários lances de escada.**1 Sim, dificulta muito
2 Sim, dificulta um pouco
3 Não, não dificulta de modo algum.

**Subir um lance de escada.**1 Sim, dificulta muito
2 Sim, dificulta um pouco
3 Não, não dificulta de modo algum.

**Curvar-se, ajoelhar-se ou dobrar-se.**1 Sim, dificulta muito
2 Sim, dificulta um pouco
3 Não, não dificulta de modo algum.

**Andar mais de 1 quilômetro.**1 Sim, dificulta muito
2 Sim, dificulta um pouco
3 Não, não dificulta de modo algum.

**Andar vários quarteirões.**
1 Sim, dificulta muito
2 Sim, dificulta um pouco
3 Não, não dificulta de modo algum.

**Andar um quarteirão.**1 Sim, dificulta muito
2 Sim, dificulta um pouco
3 Não, não dificulta de modo algum.

**Tomar um banho ou vestir-se.**1 Sim, dificulta muito
2 Sim, dificulta um pouco
3 Não, não dificulta de modo algum.

**4. Durante as últimas 4 semanas, você teve algum dos seguintes problemas com o seu trabalho ou com alguma atividade diária regular, como consequência de sua saúde física (circule uma alternativa para cada atividade)?**

**Você diminuiu a quantidade de tempo que dedicava ao seu trabalho ou a outras atividades?**1 Sim
2 Não

**Realizou menos tarefas do que você gostaria?**1 Sim
2 Não

**Esteve limitado(a) no seu tipo de trabalho ou em outras atividades?**1 Sim
2 Não

**Teve dificuldade de fazer seu trabalho ou outras atividades (por exemplo: necessitou de um esforço extra)?**1 Sim
2 Não

**5. Durante as últimas 4 semanas você teve algum dos seguintes problemas com o seu trabalho ou outra atividade regular diária como consequência de algum problema emocional (como sentir-se deprimido ou ansioso — circule uma alternativa para cada atividade)?**

**Você diminuiu a quantidade de tempo que dedicava ao seu trabalho ou a outras atividades?**1 Sim
2 Não

**Realizou menos tarefas do que você gostaria?**1 Sim
2 Não

**Não trabalhou ou não fez qualquer das atividades com tanto cuidado como geralmente faz?**1 Sim
2 Não

**6. Durante as últimas 4 semanas, de que maneira sua saúde física ou problemas emocionais interferiram nas suas atividades sociais normais, em relação à família, vizinhos amigos e em grupo (circule uma alternativa)?**1 De forma nenhuma
2 Ligeiramente
3 Moderadamente
4 Bastante
5 Extremamente

**7. Quanta dor no corpo você teve durante as últimas 4 semanas (circule uma alternativa)?**1 Nenhuma
2 Muito leve
3 Leve
4 Moderada
5 Grave
6 Muito grave

**8. Durante as últimas 4 semanas, quanto a dor interferiu com o seu trabalho normal (incluindo tanto o trabalho fora de casa como dentro de casa — circule uma alternativa)?**1 De maneira alguma
2 Um pouco
3 Moderadamente
4 Bastante
5 Extremamente

**9. Estas questões são sobre como você se sente e como tudo tem acontecido com você durante as últimas 4 semanas. Para cada questão, por favor dê uma resposta que mais se aproxime de maneira como você se sente, em relação às últimas 4 semanas.**

**Quanto tempo você tem se sentindo cheio de vigor, de vontade, de força?**

1 Todo o tempo

2 A maior parte do tempo

3 Uma boa parte do tempo

4 Alguma parte do tempo

5 Uma pequena parte do tempo

6 Nunca

**Quanto tempo você tem se sentido uma pessoa muito nervosa?**

1 Todo o tempo

2 A maior parte do tempo

3 Uma boa parte do tempo

4 Alguma parte do tempo

5 Uma pequena parte do tempo

6 Nunca

**Quanto tempo você tem se sentido tão deprimido que nada pode animá-lo?**

1 Todo o tempo

2 A maior parte do tempo

3 Uma boa parte do tempo

4 Alguma parte do tempo

5 Uma pequena parte do tempo

6 Nunca

**Quanto tempo você tem se sentido calmo ou tranqüilo?**

1 Todo o tempo

2 A maior parte do tempo

3 Uma boa parte do tempo

4 Alguma parte do tempo

5 Uma pequena parte do tempo

6 Nunca

**Quanto tempo você tem se sentido com muita energia?**

1 Todo o tempo

2 A maior parte do tempo

3 Uma boa parte do tempo

4 Alguma parte do tempo

5 Uma pequena parte do tempo

6 Nunca

**Quanto tempo você tem se sentido desanimado ou abatido?**

1 Todo o tempo

2 A maior parte do tempo

3 Uma boa parte do tempo

4 Alguma parte do tempo

5 Uma pequena parte do tempo

6 Nunca

**Quanto tempo você tem se sentido esgotado?**

1 Todo o tempo

2 A maior parte do tempo

3 Uma boa parte do tempo

4 Alguma parte do tempo

5 Uma pequena parte do tempo

6 Nunca

**Quanto tempo você tem se sentido uma pessoa feliz?**

1 Todo o tempo

2 A maior parte do tempo

3 Uma boa parte do tempo

4 Alguma parte do tempo

5 Uma pequena parte do tempo

6 Nunca

**Quanto tempo você tem se sentido cansado?**

1 Todo o tempo

2 A maior parte do tempo

3 Uma boa parte do tempo

4 Alguma parte do tempo

5 Uma pequena parte do tempo

6 Nunca

**10. Durante as últimas 4 semanas, quanto de seu tempo a sua saúde física ou problemas emocionais interferiram com as suas atividades sociais (como visitar amigos, parentes, etc)?**

1 Todo o tempo

2 A maior parte do tempo

3 Uma boa parte do tempo

4 Alguma parte do tempo

5 Uma pequena parte do tempo

**11. O quanto verdadeiro ou falso é cada uma das afirmações para você?**

**Eu costumo obedecer um pouco mais facilmente que as outras pessoas**

1 Definitivamente verdadeiro

2 A maioria das vezes verdadeiro

3 Não sei

4 A maioria das vezes falso

5 Definitivamente falso

**Eu sou tão saudável quanto qualquer pessoa que eu conheço**

1 Definitivamente verdadeiro

2 A maioria das vezes verdadeiro

3 Não sei

4 A maioria das vezes falso

5 Definitivamente falso

**Eu acho que a minha saúde vai piorar**

1 Definitivamente verdadeiro

2 A maioria das vezes verdadeiro

3 Não sei

4 A maioria das vezes falso

5 Definitivamente falso

**Minha saúde é excelente**

1 Definitivamente verdadeiro

2 A maioria das vezes verdadeiro

3 Não sei

4 A maioria das vezes falso

5 Definitivamente falso

**Ware JE, Sherbourne CD: The MOS 36 Item Short-Form Health Survey (SF-36). I. Conceptual framework and item selection. Med Care 30: 473-483, 1992.**

**Anexo 6
Índice de WOMAC**

**Seção A: Intensidade da dor**

**Quanta dor você tem?
1. Caminhando numa superfície plana.**□ Nenhuma □ Leve □ Moderada
□ Forte □ Muito Forte

**2. Subindo ou descendo escadas.**□ Nenhuma □ Leve □ Moderada
□ Forte □ Muito Forte

**3. À noite, deitado(a) na cama.**□ Nenhuma □ Leve □ Moderada
□ Forte □ Muito Forte

**4. Sentado(a) ou deitado(a).**□ Nenhuma □ Leve □ Moderada
□ Forte □ Muito Forte

**5. Ficando em pé.**□ Nenhuma □ Leve □ Moderada
□ Forte □ Muito Forte

**Seção B: Intensidade da rigidez articular**

**1. Qual a intensidade da sua rigidez logo após acordar pela manhã?**□ Nenhuma □ Leve □ Moderada
□ Forte □ Muito Forte

**2. Qual a intensidade da rigidez após sentar-se, deitar-se ou descansar durante o dia?**
□ Nenhuma □ Leve □ Moderada
□ Forte □ Muito Forte

**Seção C: Atividade física**

**Qual é o grau de dificuldade que você tem?
1. Descendo escadas.**□ Nenhuma □ Leve □ Moderada
□ Forte □ Muito Forte

**2. Subindo escadas.**□ Nenhuma □ Leve □ Moderada
□ Forte □ Muito Forte

**3. Levantando-se de uma cadeira?**□ Nenhuma □ Leve □ Moderada
□ Forte □ Muito Forte

**4. Ficando em pé?**□ Nenhuma □ Leve □ Moderada
□ Forte □ Muito Forte

**5. Curvando-se para tocar o chão?**□ Nenhuma □ Leve □ Moderada
□ Forte □ Muito Forte

**6. Caminhando no plano?**□ Nenhuma □ Leve □ Moderada
□ Forte □ Muito Forte

**7. Entrando ou saindo do carro?**□ Nenhuma □ Leve □ Moderada
□ Forte □ Muito Forte

**8. Fazendo compras?**□ Nenhuma □ Leve □ Moderada
□ Forte □ Muito Forte

**9. Colocando as meias / meias-calças?**□ Nenhuma □ Leve □ Moderada
□ Forte □ Muito Forte

**10. Levantando-se da cama?**□ Nenhuma □ Leve □ Moderada
□ Forte □ Muito Forte

**11. Tirando as meias / meias-calças?**□ Nenhuma □ Leve □ Moderada
□ Forte □ Muito Forte

**12. Deitando-se na cama?**
□ Nenhuma □ Leve □ Moderada
□ Forte □ Muito Forte

**13. Entrando ou saindo do banho?**□ Nenhuma □ Leve □ Moderada
□ Forte □ Muito Forte

**14. Sentando-se?**□ Nenhuma □ Leve □ Moderada
□ Forte □ Muito Forte

**15. Sentando-se ou levantando-se do vaso sanitário?**□ Nenhuma □ Leve □ Moderada
□ Forte □ Muito Forte

**16. Fazendo tarefas domésticas pesadas?**□ Nenhuma □ Leve □ Moderada
□ Forte □ Muito Forte

**17. Fazendo tarefas domésticas leves?**□ Nenhuma □ Leve □ Moderada
□ Forte □ Muito Forte

**Bellamy N, Buchanan WW, Goldsmith CH, Campbell J, Stitt LW. Validation study of WOMAC: a health status instrument for measuring clinically important patient relevant outcomes to antirheumatic drug therapy in patients with osteoarthritis of the hip or knee. The Journal of Rheumatology. 1988;15(12):1833-184.**

**Anexo 7**

**“Geriatric Pain Measure”**

**Por favor, responda cada pergunta, marcando-a:**

**1. Você tem ou acha que teria dor com atividades intensas como correr, levantar objetos pesados ou participar de atividades que exigem esforço físico?**

( ) não ( ) sim

**2. Você tem ou acha que teria dor com atividades moderadas como mudar uma mesa pesada de lugar, usar um aspirador de pó, fazer caminhadas ou jogar bola?**

( ) não ( ) sim

**3. Você tem ou acha que teria dor quando levanta ou carrega sacola de compras?**

( ) não ( ) sim

**4. Você tem ou acha que teria dor se subisse um andar de escadas?**

( ) não ( ) sim

**5. Você tem ou teria dor se subisse apenas alguns degraus de uma escada?**

( ) não ( ) sim

**6. Você tem ou teria dor quando anda mais de um quarteirão?**

( ) não ( ) sim

**7. Você tem ou teria dor quando anda um quarteirão ou menos?**

( ) não ( ) sim

**8. Você tem ou teria dor quando toma banho ou se veste?**

( ) não ( ) sim

**9. Você já deixou de trabalhar ou fazer atividades por causa de dor?**

( ) não ( ) sim

**10. O trabalho ou suas atividades já exigiram um esforço por causa de dor?**

( ) não ( ) sim

**11. Você tem diminuído o tipo de trabalho ou outras atividades que faz devido à dor?**

( ) não ( ) sim

**12. O trabalho ou suas atividades já exigiram muito esforço por causa da dor?**

( ) não ( ) sim

**13. Você tem problema para dormir devido à dor?**

( ) não ( ) sim

**14. A dor impede que você participe de atividades religiosas?**

( ) não ( ) sim

**15. A dor impede que você participe de qualquer outra atividade social ou recreativa (além de serviços religiosos)?**

( ) não ( ) sim

**16. A dor te impede ou impediria de viajar ou usar transportes comuns?**

( ) não ( ) sim

**17. A dor faz você sentir fadiga ou cansaço?**

( ) não ( ) sim

**18. Você depende de alguém para te ajudar por causa da dor?**

( ) não ( ) sim

**19. Na escala de 0 a 10, com 0 significando sem dor e o 10 significando a pior dor que você possa imaginar, como está a sua dor hoje?**

0 1 2 3 4 5 6 7 8 9 10

**20. Nos últimos sete dias, numa escala de zero a dez, com zero significando dor nenhuma e dez significando a pior dor que você possa imaginar, como está a sua dor hoje?**

0 1 2 3 4 5 6 7 8 9 10

**21. Você tem dor que nunca some por completo?**

( ) não ( ) sim

**22. Você tem dor todos os dias?**

( ) não ( ) sim

**23. Você tem dor várias vezes por semana?**

( ) não ( ) sim

**24. Durante os últimos sete dias, a dor fez você se sentir triste ou depressivo?**

( ) não ( ) sim

**PONTUAÇÃO: Dê um ponto para cada “SIM” e some as respostas numéricas.**

**PONTUAÇÃO TOTAL (0 – 42): __________**

**Pontuação ajustada (Pontuação Total X 238) (0 – 100): __________**

**Ferrell BA, Stein WM, Beck JC. The Geriatric Pain Measure: validity, reliability and factor analysis. J Am Geriatr Soc. 2000; 48(12):1669-1673.**

**Anexo 8
Modelo do termo de consentimento livre e esclarecido**

**Termo de Consentimento Livre e Esclarecido***(em 2 vias, uma para o participante e outra para o pesquisador)*

COMPARAÇÃO ENTRE OZÔNIO INTRA-ARTICULAR E PLACEBO NO TRATAMENTO DA ARTRITE DO JOELHO

O(a) sr(a). está sendo convidado(a) para participar de uma pesquisa com a finalidade de se obter um tratamento melhor da artrite de joelho na população idosa. Sua participação é muito importante para que esta pesquisa possa ser realizada e possamos tratar melhor da artrite do joelho em idosos, a qual leva a várias limitações e incapacidades. Neste estudo, injetaremos um gás chamado ozônio ou ar no joelho de pacientes com artrite e com idade entre 60 e 75 anos.

Para esse estudo, o(a) sr(a). está sendo convidado para uma consulta médica, na qual serão aplicados 6 questionários pelo médico pesquisador e, logo após o seu consentimento, será aplicado, no seu joelho doente, ozônio ou ar. O(a) sr(a). receberá o total de 8 injeções de ozônio ou de ar. Após a quarta e a oitava aplicação e, 4 meses depois de terminarem as aplicações, o(a) sr(a). responderá novamente os 6 questionários que serão aplicados novamente pelo médico pesquisador.

O pesquisador é médico do Ambulatório do Instituto de Geriatria e Gerontologia (IGG) da EPM-UNIFESP.

O desconforto que o(a) sr(a). poderá ter está relacionado à injeção do anestésico (infecção, reação alérgica e fraqueza, tontura, alteração da visão e da fala) e do ozônio (febre e dor local).

O(a) sr(a). não terá qualquer custo financeiro com exames e consultas realizadas.Também não haverá compensação financeira pela sua participação. Os resultados estarão à sua disposição a qualquer momento e será garantido o sigilo dessas informações. Caso o(a) sr(a). decida deixar de participar do estudo, não haverá qualquer prejuízo à continuidade de seu tratamento na instituição.

Em qualquer etapa do estudo, o(a) sr(a). poderá esclarecer dúvidas com a equipe médica da pesquisa (Dr. Carlos César Lopes de Jesus, Dra. Virgínia Fernandes Moça Trevisani e Dra. Fânia Cristina dos Santos à rua Francisco de Castro, 105 - tel. 5575-4848) ou com o Comitê de Ética em Pesquisa (rua Botucatu, 572 - 1º andar, conjunto 14, tel 5571-1062). Neste projeto o pesquisador principal é o Dr. Carlos César Lopes de Jesus.

Eu,_______________________________________, declaro ter sido suficientemente informado a respeito desta pesquisa que busca melhorar o tratamento da dor do joelho da população idosa com artrite. Discuti com o Dr. Carlos César Lopes de Jesus sobre a minha decisão em participar, tendo ficado claro os propósitos do estudo, seus desconfortos e riscos, a garantia de atendimento médico, caso necessário, de confidencialidade e de esclarecimentos permanentes. Sei que a minha participação é isenta de custos ou compensação financeiros. Concordo em participar voluntariamente e poderei retirar meu consentimento a qualquer momento, sem penalidades no meu atendimento neste serviço.

Data: ____/____/____

Assinatura do paciente ou do representante legal:

__________________________________________________________

Data: ____/____/____

Assinatura do responsável pela leitura do Termo de Consentimento:

_________________________________________________________

**Anexo 9**

**Modelo do Termo de Retirada do Consentimento**É garantida a liberdade da retirada do consentimento para que o paciente deixe de participar do estudo, sem quaisquer tipos de prejuízo ao indivíduo.

Eu ______________________________________________ revogo o consentimento prestado no dia _______________ e afirmo que não desejo prosseguir no estudo que me foi proposto, o qual dou como finalizado nesta data. Cidade: _________________________________________ Data: _________________

Assinatura do pesquisador: _______________________________________________
Assinatura do participante: _______________________________________________
Testemunha:

**Anexo 10
Termo de responsabilidade e compromisso do pesquisador responsável**

**_______________________________________________________________________**
Eu, Carlos César Lopes de Jesus, pesquisador responsável pelo projeto “Comparação entre ozônio intra-articular e placebo no tratamento da artrite do joelho”, declaro estar ciente e que cumprirei os termos da Resolução 196 de 09/10/96 do Conselho Nacional de Saúde do Ministério da Saúde e declaro:

1. Assumir o compromisso de zelar pela privacidade e sigilo das informações;
2. Tornar os resultados desta pesquisa públicos sejam eles favoráveis ou não; e
3. Comunicar ao Comitê de Ética em Pesquisa da Escola Paulista de Medicina — Universidade Federal de São Paulo (EPM — UNIFESP) sobre qualquer alteração no projeto de pesquisa, nos relatórios semestrais ou por meio de comunicação protocolada, que me forem solicitadas.

São Paulo, 26 de julho de 2010.
__________________________________________________
Carlos César Lopes de Jesus
Pesquisador
**_______________________________________________________________________**

**Anexo 11
Declaração de condições da instituição**

**_______________________________________________________________________**
Eu, Álvaro Nagib Atallah, chefe do Departamento de Medicina Interna e Medicina Baseada em Evidências da Escola Paulista de Medicina – Universidade Federal de São Paulo (EPM – UNIFESP), declaro estar ciente dos termos da Resolução 196 de 09/10/96 do Conselho Nacional de Saúde do Ministério da Saúde e que o Departamento de Saúde Integral e Comunitária possui recursos e infra-estrutura para atender eventuais problemas resultantes do projeto de pesquisa “Comparação entre o ozônio intra-articular e o placebo no tratamento da artrite de joelho”.

São Paulo, 26 de julho de 2010.
__________________________________________________
Prof. Dr. Álvaro Nagib Atallah
Chefe do Departamento de Medicina Interna e Medicina Baseada em Evidências
Escola Paulista de Medicina – Universidade Federal de São Paulo (EPM – UNIFESP)
**_______________________________________________________________________**

**Anexo 12
Currículo Lattes dos pesquisadores**

**Orientadora**Profa. Dra. Virgínia Fernandes Moça Trevisani
*http://lattes.cnpq.br/9054730236021091*

**Co-orientadora**Profa. Dra. Fânia Cristina dos Santos
*http://lattes.cnpq.br/9874664960025710*

**Pesquisador**Carlos César Lopes de Jesus
*http://lattes.cnpq.br/5070916128868023*

**Dados pessoais
Nome**Carlos César Lopes de Jesus

**Nome em citações bibliográficas**Lopes de Jesus, Carlos César

**Sexo**Masculino

**Endereço profissional**Escola Paulista de Medicina — Universidade Federal de São Paulo — Disciplina de Medicina Interna e Terapêutica / Centro Cochrane do Brasil
Rua Botucatu, 740 – Vila Clementino – São Paulo
Fone: +55 (11)5576-4203

**Endereço eletrônico**caceloje@gmail.com

Bibliografia consultada

Rother ET, Braga MER. **Como elaborar sua tese: estrutura e referências.** São Paulo: [s.n.]; 2005. 122 p.
