# Supplementary material for: Comparison between intra-articular ozone and placebo in the treatment of knee osteoarthritis: A randomized, double-blinded, placebo-controlled study
Source: PLoS One. 2017 Jul 24;12(7):e0179185. doi: 10.1371/journal.pone.0179185 (PMC5524330; doi:10.1371/journal.pone.0179185)
Supplement: S3 File — (DOC) [file pone.0179185.s003.doc]

Carlos César Lopes de Jesus
**Comparison between intra articular ozone and a placebo in the treatment of knee osteoarthritis**

**Sao Paulo**2010

**Cataloguing data**

Lopes de Jesus, Carlos César
**Comparison between intra articular ozone and a placebo in the treatment of knee osteoarthritis / Carlos César Lopes de Jesus, Sao Paulo, 2010. 55 pages.**

Thesis (Doctorate degree) — Federal University of Sao Paulo. Paulista School of Medicine. Post-Graduation Program in Internal Medicine and Therapeutics.

1. Osteoarthritis; 2. Placebo; 3. Intra articular ozone / therapeutic use; 4. Randomized, double-blinded clinical trial; 5. Results from treatment; 6. Effectiveness.

Carlos César Lopes de Jesus
**Comparison between intra articular ozone and a placebo in the treatment of knee osteoarthritis**

Research project to be developed at Paulista School of Medicine – Federal University of Sao Paulo to obtain the title of Doctor in Sciences

**Sao Paulo**2010

Index

**List of abbreviations, acronyms, symbols and signals** 06

***Abstract*** 07

**General informations** 09

**Research Project** 11

**1 Reasons and objectives of research** 11
1.1 Context 11
1.2 Hypothesis 15
1.3 Objectives 15

**2 Work plan and Methods** 16
2.1 Type of study 16
2.2 Locals 16
2.3 Sample 16
2.4 Conducting the study 16
2.5 Selection 16
2.6 Technique 17

**3 Variables** 19
3.1 Primary variable 19
3.2 Additional data 19
3.3 Statistical method 19
3.4 Results 20

**4 Research steps** 21

**5 Schedule** 22

**6 List of required materials** 23

**7 Budget** 24

**8 Research monitoring** 27

**9 Risks and benefits** 28

**10 Property of information and research dissemination** 29

**Bibliography** 30

**Tables** 31 1 Radiological criteria 31 2 Diagnostic criteria of idiopathic knee osteoarthritis 32

**Attachments** 33 1 Model of Data Collection Form 33 2 Visual Analogic Scale (VAS) 34
3 Lequesne Functional Index 35
4 Timed Up and Go Test (TUG) 37
5 SF-36 38
6 WOMAC 44
7 Geriatric Pain Measure 47
8 Written Informed Consent Form 49
9 Term of Withdrawal of Written Consent Form 51
10 Term of Responsibility and Commitment of the Responsible Researcher 52
11 Statement of institutional conditions 53
12 Researchers’ curriculum lattes 54

**Consulted bibliography** 55

List of abbreviations, acronyms, symbols and signals

**EPM-UNIFESP** Paulista School of Medicine – Federal University of Sao Paulo

**LLC** Limited Liability Company

**AVS** Analogic Visual Scale

**TUG** Timed Up and Go Test

**SF-36** Medical Outcomes Study 36 – Item Short – Form Health Survey

**WOMAC** Western Ontario and McMaster Universities Osteoarthritis Index

**GPM** Geriatric Pain Measure

**%** Percentage

**α** Alpha error

**β** Beta error

**http** Hypertext Transfer Protocol

**OA** Ostearthritis

**O3** Ozone

**AIDS** Acquired Immunodeficiency Syndrome

**µg/ml** Micrograms per milliliter

**cm** Centimeter

**°GL** Degrees Gay-Lussac

**ml** Mililiter

**H0** Nullity hypothesis

**P** Percentage of events

**H1** Alternative hypothesis

**A4** 210mm by 297mm paper size

**g/cm²** Grams per square centimeter

**www** World Wide Web

*Abstract*

**Title**Comparison between intra articular ozone and a placebo in the treatment of knee osteoarthritis

**Author**Carlos César Lopes de Jesus

**Institution**Internal Medicine and Therapeutics Discipline

Paulista School of Medicine - Sao Paulo Federal University (EPM – UNIFESP)

Rua Botucatu, 740 – 3º andar

Vila Clementino - Sao Paulo, SP

Brazil

Zip Code: 04023-900

Phone: +55(11) 5576-4203

e-mail: [caceloje@gmail.com](mailto:caceloje@gmail.com)

Curriculum lattes: <http://lattes.cnpq.br/5070916128868023>

**Context**Osteoarthritis is the most common of all articular diseases. Its importance comes from its economic impact concerning productivity (main cause of lost working days) as well as the cost of treatment (chronic use of analgesics, anti-inflammatory agents, condroprotectors and disease-modifying drugs). Although the etiology of such sickness is not yet clearly understood, it has been verified that osteoarthritis is a family of diseases that has the cartilage as the target organ, in which certain biomechanical factors play a central role and that has certain risk factors (as age, weight and profession) being yet of primordial importance. As nowadays there is no treatment capable of preventing or improving the basic morbid process, clinical treatment aims mainly to relieve pain. This way, it is of vital importance to answer the question: knee osteoarthritis treatment with intra articular ozone is more effective than the treatment of knee osteoarthritis with intra articular placebo?

**Objective**To determine if the treatment of knee osteoarthritis with intra articular ozone is more effective than the treatment of knee osteoarthritis with an intra articular placebo. Research hypothesis is that the treatment of knee osteoarthritis with ozone is 30% more effective than the treatment of knee osteoarthritis with a placebo.

**Design**Multicenter, comparative, randomized, double-blind clinical trial.

**Setting**Outpatients Department of Geriatrics and Gerontology Discipline of Paulista School of Medicine Federal University of São Paulo, Pró-Vida – Center for Total Health Assistance LLC and Outpatients Department of Rheumatology Discipline - Santo Amaro University – School of Medicine, São Paulo, SP, Brazil.

**Sample**Patients with knee osteoarthritis from Outpatients Department of Geriatrics and Gerontology Discipline of Paulista School of Medicine Federal University of São Paulo (EPM – UNIFESP), Pró-Vida – Center for Total Health Assistance LLC and Outpatients Department of Rheumatology Discipline - Santo Amaro University – School of Medicine.

**Exclusion criteria**

Patients aged less than 60 years or more than 85 years, those who not give their permission to be included in the study, patients with mental and / or neurologic deficit, patients with recent knee traumas or with suspect of an associated knee lesion, and patients with coxofemoral articulation affections.

**Main outcome**Efficacy of intra articular use of ozone or placebo in the treatment of knee osteoarthritis clinically and radiologically diagnosed according to the American College of Rheumatology criteria.

Efficacy will be measured in accordance with: 1) Analogic Visual Scale (AVS); 2) Lequesne Functional Index; 3) Timed Up and Go Test (TUG); 4) Medical Outcomes Study 36 – Item Short – Form Health Survey (SF-36); 5) Western Ontario and McMaster Universities Osteoarthritis Index (WOMAC) and Geriatric Pain Measure (GPM).

**Statistical methods**The sample size was estimated in 96 individuals, considering a pain reduction of 30% in placebo group and a significant difference of 30% in favor of the treatment group.

It was established an α = 0,05 and a β = 0,20.

**Headings**1. Osteoarthritis; 2. Placebo; 3. Intra articular ozone / therapeutical use; 4. Randomized double-blind clinical trial; 5. Treatment result; 6. Effectivness.

General Information

**Places where the study will be developed**

1.Outpatients Department – Geriatrics and Gerontology Discipline

Paulista School of Medicine – Federal University of Sao Paulo (EPM – UNIFESP)
Rua Prof. Francisco de Castro, 105

CEP: 04025-001

Sao Paulo – SP

Brasil
Telephone: +55 (11) 5575-4848

2. Pró-Vida – Center for Total Health Assistance LLC
Av. Paes de Barros, 411 – cj. 14 e 15

CEP: 03115-020

Sao Paulo – SP

Brasil
Telephone: +55 (11) 2307-6202

3. Outpatients Department – Rheumatology Discipline

University of Santo Amaro – Medicine College
Rua Cássio de Campos Nogueira, 2031

CEP: 04829-310

Sao Paulo – SP

Brasil
Telephone: +55 (11) 2141-8813

**Main researcher**Carlos César Lopes de Jesus, <[caceloje@gmail.com](mailto:faniacs@uol.com.br)>

Master in Health Sciences

Doctor’s degree student at Internal Medicine and Therapeutics Discipline

Paulista School of Medicine – Federal University of Sao Paulo (EPM – UNIFESP)

Sao Paulo, SP

Brazil

Telephone: +55 (11) 5576-4203
Curriculum lattes: <http://lattes.cnpq.br/5070916128868023>

**Conflict of interests**No one.

**Funding**No one.

**Adviser**Virgínia Fernandes Moça Trevisani, Master, PhD, <vmoca@uol.com.br>

Adviser of Internal Medicine and Therapeutics

Paulista School of Medicine – Federal University of Sao Paulo (EPM – UNIFESP)

Sao Paulo, SP

Brazil
Telephone: +55 (11) 5576-4203
Curriculum lattes: http://lattes.cnpq.br/9054730236021091

**Second reader**Fânia Cristina dos Santos, Master, PhD, <faniacs@uol.com.br>

Chief of Outpatients Department of Geriatrics and Gerontology Discipline

Paulista School of Medicine – Federal University of Sao Paulo (EPM – UNIFESP) Coordinator of Pain Group of Geriatrics and Gerontology Discipline

Paulista School of Medicine – Federal University of Sao Paulo (EPM – UNIFESP) Coordinator of Elderly Pain Committee of Brazilian Society for the Study of Pain

Sao Paulo, SP

Brazil
Telephone: +55 (11) 5575-4848
Curriculum lattes: http://lattes.cnpq.br/9874664960025710

**Research title**Comparison between intra articular ozone and placebo in the treatment of knee osteoarthritis

**Objectives**To determine if treatment of knee osteoarthritis with intra articular ozone is more effective than the treatment with a placebo concerning pain control and functional and quality of life improvement.

**Estimated cost**R$ 6,079.80 (USD$ 1,882.29)

Research Project

1 Reasons and objectives of research **1.1 Context**Osteoarthritis (OA) is a disease of the diarthrodial joints; clinically characterized by pain and functional limitation; - radiographically by osteophytes and narrowing of the joint space; and histopathologically by alterations in the integrity of the cartilage. Being the commonest of all the joint diseases, its importance also encompasses its economic impact, in terms of both productivity (main isolated cause of lost days of work) and cost of the treatment (chronic use of painkillers, anti-inflammatory agents, chondroprotective drugs, and disease modifying drugs). Although the etiology of this disease is not been clearly understood, it has been noted that OA is a disease whose target organ is the cartilage, that certain biomechanical factors play a central role, together with factors of risk (such as age, weight, and profession) of primordial importance. As there is no treatment able to prevent or lessen the basic morbid process, clinical treatment aims mainly to relieve the pain, with orthopaedic interventions being reserved for situations that cannot be controlled with more conservative therapy (1).

**Ozone**The distinctive smell of ozone was registered for the first time in 1785 by Van Mauran, but this gas would only be "discovered" in 1840 by the German chemist Christian Frederick Schönbein, in the University of Basel, Switzerland. He decided to give it the name of ozone (from the Greek, smell) due to the strong odor of the substance (2, 3).

Ozone is a form of oxygen that occurs naturally in the atmosphere. It is created in nature through the action of ultraviolet rays on oxygen. Ozone is formed by the action of electric unloadings in oxygen and is also produced commercially in generators which release an electric unloading on a special condenser containing oxygen (2, 3).

Due to the fact that ozone has three atoms of oxygen, it is known chemically as O_3_ (2, 3).

**Ozone in Medicine**Used mainly to kill viruses, destroy bacteria, and eliminate fungi, ozone produces important benefits to the human body, such as oxygenation of the blood, the improvement in blood circulation, and the facilitation of liberation of oxygen for the tissues. It is also an important immunological regulator. For this reason, the number of health problems that can respond favorably to treatment with ozone is quite broad. Physicians have been using ozone in areas such as angiology, dermatology (including allergies), gastroenterology, intensive therapy, gynecology, neurology, dentistry, oncology, orthopedy, proctology, radiology, rheumatology, surgery (including vascular surgery) and urology (2, 3).

According to the Medical Society for Ozone of Europe and the National Center of Scientific Inquiry of Cuba, physicians use ozone in the following diseases: abscesses, acne, AIDS, allergies (hypersensitivity), anal cracks, arthritis, osteoarthritis, asthma, neoplasms, cerebral sclerosis, circulatory disturbances, liver cirrhosis, climacteric syndrome, obstipation, cornea ulcer, cystitis, decubitus ulcers, diarrhea, fistulas, diseases caused by fungi, boils, gangrene, gastroduodenal ulcers, gastrointestinal disturbances, giardiasis, glaucoma, hepatitis, simple herpes and shingles, hypercholesterolemia, ulcerative colitis, mycosis, nervous disturbances, osteomyelitis, Parkinson’s disease, polyarthritis, Raynaud’s syndrome, retinitis pigmentosa, rheumatoid arthritis, scars (after radiation), senile dementia, septicemia, sinusitis, spondylitis, stomatitis, Sudeck’s syndrome (post-traumatic osteoporosis), thrombophlebitis, ulcer in the lower limbs, vulvovaginities, and injuries (2, 3).

**Ozone in Dentistry**According to the German dentist Fritz Kramer, ozone presents the following applications:

- in the form of ozonized water it can be used in the treatment of gums, periodontitis, ulcers, or stomatities;
- in the form of spray to clean the affected area, disinfect the buccal mucous membrane and cavities, and in odontologic surgeries;
- in the form of a jet to clean teeth cavities to be filled or subjected to canal treatment (2, 3).

**How ozone treatment is applied?**In the last sixty years, more than a dozen methods have been developed for the application of ozone in medical therapies. In most cases, small quantities of ozone are added to pure oxygen (generally 0.05 parts of ozone to 99.95 parts of oxygen for internal use and 5 parts of ozone to 95 parts of oxygen for external applications). The right quantity depends on each case, since a small quantity of ozone can be inefficient and a great quantity can block immunological function (2, 3).

Ozone can be administered by the rectal route, intramuscular route, through major and minor hemotherapy, external use of ozonized water, local administration of the gas (using a covering of special plastic), or using ozonized oil in dermatological affections (2, 3).

**Contra-indications**

Acute alcoholic intoxication, recent heart attack, hemorrhage of any organ, pregnancy, hyperthyroidism, thrombocytopenia and allergy to ozone constitute contra-indications to the use of ozone (4).

**Mechanisms of action**Ozone has anti-inflammatory effects (due to its influence on prostaglandins and peroxidases), and acts as a broad painkiller, antiseptic, and germicide. Furthermore, it modulates biological responses, shown through a tendency to normalization of glucose and other blood metabolites (2, 3).

It is believed that the possible mechanism of the action of ozone is linked to the generation of secondary products. It is conceived that the formed organic compounds, such as the organic peroxide ozonides, have different biological actions that provide a set of therapeutic properties to the ozone, for example, improvement in tissue oxygenation, immunological modulation, modulation of the liberation of certain autacoids, germicidal power, and the capacity for metabolic regulation (2, 3).

Nevertheless, one of the most important properties of ozone is its antioxidant effect, which takes place by means of the stimulation of the enzymes pertaining to the antioxidant system of the organism (5)

**Side effects**In recent years, the role of the concentration of ozone as well as the duration of the effects have been studied according to four parameters: the extension of the hemolysis, intra-erythrocytic reduced glutathione, viability of mononuclear cells of the blood, and production of cytokines. It has already been demonstrated that ozone has opposing effects. Ozone in high concentrations damages the immune response of cells and of liquids in animals and in individuals who have undergone chronic treatment. In fact, when high concentrations of ozone are used (above 78µg/ml) and especially when blood is exposed to constant ozone insufflation for periods greater than 30 seconds, there is progressive intensification of hemolysis, which reaches 52%, a reduction in the levels of reduced glutathione in the intra-erythrocytic environment (of up to 47%), a significant reduction in the viability of the mononuclear cells of the blood, and erratic production of cytokines. On the other hand, when the contact of the ozone with the blood lasts only a few seconds and the concentration of ozone is inferior to 78µg/ml of blood, hemolysis does not surpass 6%, the levels of reduced glutathione in the intra-erythrocytic environment fall to 8.3%, the viability of the blood mononuclear cells of the blood is not altered, and there is a significant production of cytokines.

These results are due, first of all, to the important antioxidant properties of plasma and also to the fact that all metabolically active cells dispose of antioxidant mechanisms, in the form of several enzymatic systems, such as catalase, superoxide dismutase, and glutathione reductase. The efficiency of the glutathione homeostasis is impressive: in at most 30 minutes after treatment with ozone, the levels of reduced glutathione in the intra-erythrocytic environment return to normal. It has already been demonstrated that glucose-6-phosphate dehydrogenase and 6-phosphogluconate dehydrogenase are basic enzymes that, while transforming glucose into ribulose-5-phosphate, produce nicotine adenine dinucleotide phosphate, the basic substrate for the cycle of oxi-reduction of glutathione. Considering the enormous area exposed by erythrocytes it is probable that the action of ozone is dispersed throughout an almost infinite number of targets of the plasmatic membrane, hardly reaching the cytoplasm, as is demonstrated by the negligible and transitory reduction in the levels of intra-erythrocytic reduced glutatihone (2, 3).

Thus, it seems clear that the potential toxicity of ozone does not obstruct its use (if the appropriate concentrations are used) since blood has the capacity to minimize the formation of free radicals and convert oxidants into less toxic variants. In the correct dose, ozone, like any other drug, can be more beneficial than harmful. In addition, the dogmatic conclusion that oxidants are always damaging is being rethought. In fact, the production of highly reactive variants in low levels may have an important role in cellular proliferation and in the defense and regulation of the immune system. The activity of nitric oxide is an example (2, 3).

In relation to the adverse reactions of intra-articular administration of ozone, Rifá and Musa observed only reactions of low complexity (immediate or delayed pain and fever), which leads us to consider the method as harmless. In their study, the most frequent side effect was intense pain with radiation to the leg or muscles with a duration of a few seconds that disappeared spontaneously. It was inferred that such pain might be due to a technique mistake since the gas is injected outside the join, into the periarticular space. The effect was generally observed in obese patients in whom the space or joint line was difficult to locate or, perhaps, due to the abundant fatty pad the needle did not penetrate the joint. The pain was related to the volume increase resulting from the injected gas (5).

**Ozone and osteoarthritis**In 1990, Cuban investigators studied 234 patients with complaints of pain and problems related to the lumbar and sacral columns, knees and other joints. A total of 20 intra-muscular injections of oxygen and ozone were administered over a period of 30 days; a daily injection during the first 10 days and another 10 injections on alternate days. All the patients were carefully examined, diagnosed, and evaluated prior to the study (2, 3).

Results were impressive: 208 patients (89 %) reported a complete disappearance of pain; 24 (10 %) reported some degree of improvement and 2 patients (1 %) reported that there were no alterations in their level of health. During the follow-up of these patients, the investigators verified the majority of patients remained free of symptoms for 3 to 6 months, whereas some did not feel pain for up to 11 months after the treatment (2, 3).

Due to improvement in methods of treatment, these results were better than those of a prior Cuban study carried out with 122 patients with osteoarthritis, in whom 71.8 % of the patients treated with ozone reported a complete disappearance of pain, whereas 21.8 % reported an improvement (6).

In the Center for Medical and Surgical Studies in Cuba, 60 patients with osteoarthritis (the majority with compromised knee joints) received one intra articular injection of ozone per week for a total of 10 weeks. Only 4 of these 60 patients experienced a return of the pain, while the majority (93.3 %) remained free of symptoms. The investigators concluded that this easily administered and low cost treatment induced the disappearance of pain after the first ozone applications, as well as restoring normal joint movement (7).

So it is relevant to answer the inquiry question: is the treatment of knee osteoarthritis with intra articular ozone more effective than the treatment of knee osteoarthritis with a placebo regarding the control of pain and improvement in function?

**1.2 Hypothesis**The hypothesis is that the treatment of knee osteoarthritis with intra articular ozone is more effective than the treatment of knee osteoarthritis with a placebo regarding the control of pain and functional and quality of life improvement.

**1.3 Objectives**To determine if the treatment of knee osteoarthritis with intra articular ozone is more effective than the treatment of knee osteoarthritis with a placebo regarding the control of pain and functional and quality of life improvement.

2 Work plan and Methods

This inquiry project will be sent for evaluation by the Committee of Ethics in Inquiry of the Paulista School of Medicine - Federal University of Sao Paulo (EPM-UNIFESP). The clinical test will begin only after approval from this Committee.

**2.1 Type of study**Multicenter, randomized, double-blinded clinical trial.

**2.2 Locals**Outpatients Department – Geriatrics and Gerontology Discipline – Paulista School of Medicine – Federal University of São Paulo (EPM – UNIFESP), Pró-Vida – Center for Total Health Assistance LLC and Outpatients Department – Rheumatology Discipline - University of Santo Amaro – School of Medicine.

**2.3 Sample
Inclusion criteria**The study will include patients with knee osteoarthritis, aged between 60 and 85 years, who give their permission to be included in the study.

**Exclusion criteria**The following patients will be excluded: those aged less than 60 years or more than 85 years, those who not give their permission to be included in the study, patients with mental and / or neurological deficiency, patients with recent knee trauma or suspicion of another associated injury, and patients who present affections of the hip joint.

**Exit criteria**

- Voluntary exit from the study;
- Absence from more than two consecutive sessions of treatment;
- Presentation of irregularity of treatment.

**2.4 Conducting the study**

The patients and relatives will be informed about the study, its risks and characteristics. The patients will only be admitted to the study after signing the Written Informed Consent Form.

**2.5 Selection**Patients will be selected using the clinical and radiological criteria of the American College of Rheumatology. The selected patients will be randomized on the basis of a chart of random numbers to receive intra articular ozone or a placebo. An integrant of the study, who is not going to take part in the evaluation of the patients, will have the role of producing ozone and giving the main researcher ozone or a placebo. He / she will be the only one who knows which treatment each patient receives. Neither the patient, nor the main researcher will know if the syringes dispensed contain ozone or a placebo as they will be identical.

**2.6 Technique**The intra articular injection will be administered with the patient sitting, legs hanging off the stretcher. The doctor will wash their hands before putting on sterile gloves. The injection will be administered in the suprappatelar bursa in order to avoid insertion of the needle between juxtaposed cartilaginous surfaces. The superior extremity of the patella will be identified and used as a landmark for the injection. The injection will be administered in the side part of the knee since it can be reached more easily. The needle will be inserted 1 to 3cm below the superior aspect of the patella and will be slightly cranially directed to penetrate the suprapatellar bursa. If it is not possible to obtain the synovial fluid, the needle will be redirected between the superior surface of the patella and the patellar groove of the femur. Another alternative will be considered in patients with a deformity that prevents extension of the knee. In these cases, the aspiration and infiltration will be achieved when the needle is inserted in an antero-posterior lateral or medial direction to the inferior patellar tendon. The needle will pass through the fat layer into the knee joint space, between the femoral condyle and the tibial plateau (8).

Antisepsis will be performed with alcohol 70ºGL; 1ml of lidocaine 2% without a vasoconstrictor will be administered with a syringe for insulin in the region of the injection in order to provide an anesthetic effect; the syringe will be connected to the route of exit of ozone and 10ml of the gas will be collected, the quantity that will be injected in the joint when advancing the needle (number 20) 2 to 3cm depending on the fatty pad, in a slightly oblique way backwards and inwards until feeling a vacuum sensation which will enable easy penetration of the gas without offering resistance; the joint will be aspirated firstly to empty any spills that may exist and verify that the syringe is not inside a blood vessel (8, 9).

A concentration of 20 μg/ml will be used; to supply this concentration in the OZONE & LIFE equipment the oxygen calibrator will be placed at 1ml and the clock of the ozone generator in position 8.

The procedure will be carried out once a week with a total of 8 sessions.

During the questioning and physical examination, data of interest will be obtained such as age, sex, predisposing factors, evolution time, previous treatment, joint mobility, and adverse reactions, among others.

In order to evaluate the efficiency of the intra articular use of ozone or a placebo in the treatment of knee osteoarthritis confirmed clinically and radiologically, according to the criteria of the American College of Rheumatology, the following tools will be used: 1) Analogic Visual Scale (AVS); 2) Lequesne Functional Index; 2) Timed Up and Go Test (TUG); 3) SF36; 5) WOMAC (Western Ontário and McMaster Universities Osteoarthritis Index) and 6) Geriatric Pain Measure (GPM).

Patients will be evaluated on inclusion in the study, after 4 sessions of treatment, after 8 sessions of treatment, and after the end of treatment.

**Sampling**

A probabilistic sample will be used, simple random sample. The names of the patients will be subjected to a simple draw without replacement, using a chart of random numbers. In this way, 96 individuals will be selected. The chart of random numbers will be produced in the section “random numbers” of an electronic spreadsheet (Microsoftware package Excel for Windows 7, Microsoftware package Inc., Redmond, WA).

**Written Informed Consent Form**The individuals for eligible the study based on the inclusion criteria will be invited to take part by the main investigator. At this moment, informations about the study will be given (objectives, risks, benefits and proceedings to which they will be subjected). After confirmation of the wish to participate voluntarily in the study, a copy of the Written Informed Consent Form will be given to the patient (attachment 7) so that he / she can read its content, understand it, and clarify any doubts. The individual will only be formalized to participate in the inquiry after signature of the Written Informed Consent Form.

3 Variables

**3.1 Primary variable**Efficiency of intra articular use of ozone in the treatment of knee osteoarthritis.

**3.2 Complementary data**

- Name
- Age
- Profession
- Weight
- Height

**3.3 Statistical method**

**Sample size calculation**
The sample size calculation was determined to guarantee the statistical power for the two primary endpoints. For these variables, a sample size of 40 evaluable patients provided an 80% power to detect a difference of efficacy of 30% between the groups, with a two-sided alpha level of 0.025 (10). Therefore, a total of 80 evaluable patients were required to analyze the primary endpoints of the study and approximately 96 patients were predefined to be randomized considering a dropout rate of about 20% (10).

**Statistical analysis**Data will be collected in a standardized form (attachment 10) and stored in an electronic data spreadsheet (Microsoftware package Excel for Windows 7. Redmond, WA, USA), in which each line will correspond to a form of data collection and each column to collected data. Two data entries will be carried out by different typists, in an independent and blinded way. Any controversies will be resolved by a consensus meeting.

The descriptive analysis will be carried out calculating the 95% confidence interval for each estimated point. The calculations will be carried out with the help of the statistical program SPSS20.0.

**Variables to be analyzed**The percentage improvement in pain and function found in the inquiry will be compared to the hypothesis of the inquiry.

**Statistical hypotheses**

- Ho:P = 30% (The improvement in pain and function of the knee with osteoarthritis is equal to 30%).
- H1:P ≠ 30% (The improvement in pain and function of the knee with osteoarthritis is different from 30%).

**Statistical tests**The Student t test will be used to perform the analysis.

**Alpha value**An alpha value (α) equal to or greater than 0.05 will be used in the statistical test to reject the null hypothesis.

**3.4 Presentation of results**Tables and graphs will be used for data presentation and alpha values presented to the 4th decimal place**.**

4 Research steps

**Research steps
Step I**: **Project of Research**Plan of work to verify if the hypothesis can be denied or not and to detail the procedures of execution and spreading of the research. Duration: 24 weeks.

**Step II: Identification and selection of the studies**Duration: 20 weeks.

**Step III: Data collection**
Attainment of the foreseen data. Duration: 20 weeks.

**Step IV: Storage of data**Registration and organization of collected data. Duration: 16 weeks.

**Step V: Tabulation of data**Tabulation and building of graphics. Duration: 12 weeks.

**Step VI: Data analysis**Attempt to evidence the relations displayed between the summaries and the variables. Duration: 12 weeks.

**Step VII: Data interpretation**The aim is to give a broader meaning to the answers, tying them to other knowledge and explication of the final results, considered relevant. Duration: 12 weeks.

**Step VIII: Final report and original article**General exposition of the research and elaboration of the original article. Duration: 16 weeks.

5 Schedule

| **Month|Year** | **Step I** | **Step II** | **Step III** | **Step IV** | **Step V** | **Step VI** | **Step VII** | **Step VIII** |
| --- | --- | --- | --- | --- | --- | --- | --- | --- |
| **02|2010** | O |  |  |  |  |  |  |  |
| **03|2010** | O |  |  |  |  |  |  |  |
| **04|2010** | O |  |  |  |  |  |  |  |
| **05|2010** | O |  |  |  |  |  |  |  |
| **06|2010** | O |  |  |  |  |  |  |  |
| **07|2010** | O |  |  |  |  |  |  |  |
| **08|2010** |  | O | O |  |  |  |  |  |
| **09|2010** |  | O | O |  |  |  |  |  |
| **10|2010** |  | O | O |  |  |  |  |  |
| **11|2010** |  | O | O |  |  |  |  |  |
| **12|2010** |  | O | O | O |  |  |  |  |
| **01|2011** |  |  | O | O |  |  |  |  |
| **02|2011** |  |  | O | O |  |  |  |  |
| **03|2011** |  |  | O | O |  |  |  |  |
| **04|2011** |  |  |  |  | O |  |  |  |
| **05|2011** |  |  |  |  | O | O | O | O |
| **06|2011** |  |  |  |  | O | O | O | O |
| **07|2011** |  |  |  |  |  | O | O | O |
| **08|2011** |  |  |  |  |  |  |  | O |

**O:** Planned | **X:** Accomplished

6 List of Required Materials

**Consumables**

| **Item** | **Material** | **Quantity** |
| --- | --- | --- |
| **01** | Blue pens | 004 |
| **02** | Xerox copies | 250 |
| **03** | Bond paper, 75g/cm², A4 | 500 |
| **04** | Color printer ink | 001 |
| **05** | Black printer ink | 001 |
| **06** | Insuline syringes (with needle) | 110 |
| **07** | Lidocaine — vial with 20ml of solution | 006 |
| **08** | 10ml syringes with needle | 110 |

**Permanent materials**

| **Item** | **Material** | **Quantity** |
| --- | --- | --- |
| **01** | Ozone generator | 001 |
| **02** | Oxygen regulator | 001 |
| **03** | Oxygen cylinder | 001 |

7 Budget

**Consumables**

**Item** Blue pen **Quantity** 04 **Value of each one** R$ 1,50 **Total value** R$ 6,00 **Justification** Used to fulfill the Data Extraction Forms of identified articles

**Item** Xerox copies **Quantity** 500 **Value of each one** R$ 0,07 **Total value** R$ 35,00 **Justification** Elaboration of the Written Informed Consent Forms and Data Extraction Forms

**Item** Bond paper A4, 75g/m² **Quantity** 500 **Value of each one** R$ 0,04 **Total value** R$ 20,00 **Justification** Used to print the Data Extraction Forms, the Partial Reports (of
 the pupil, the adviser and the second reader) and the Final
 Reports (of the pupil, the adviser and the second reader)

**Item** Black printer ink  **Quantity** 01 **Value of each one** R$ 92,00 **Total value** R$ 92,00 **Justification** Used to print the Data Extraction Forms, the Partial Reports (of
 the pupil, the adviser and the second reader) and the Final
 Reports (of the pupil, the adviser and the second reader)

**Item** Color printer ink **Quantity** 01 **Value of each one** R$ 116,00 **Total value** R$ 116,00 **Justification** Used to print the Data Extraction Forms, the Partial Reports (of
 the pupil, the adviser and the second reader) and the Final
 Reports (of the pupil, the adviser and the second reader)

**Item** Insulin syringes with needle **Quantity** 110 **Value of each one** R$ 0,60 **Total value** R$ 66,00 **Justification** Used in the treatment of knee osteoarthritis

**Item** Lidocaine vial with 20ml of solution **Quantity** 06 **Value of each one** R$ 5,80 **Total value** R$ 34,80 **Justification** Used for analgesia in the treatment of knee osteoarthritis

**Item** 10 ml syringes with needle  **Quantity** 110 **Value of each one** R$ 1,00 **Total value** R$ 110,00 **Justification** Used in the treatment of knee osteoarthritis

**Permanent materials**

**Item** Ozone generator **Quantity** 01 **Value** R$ 500,00 **Justification** Used in the treatment of knee osteoarthritis

**Item** Oxygen regulator  **Quantity** 01 **Value** R$ 500,00 **Justification** To regulate the flux of oxygen in ozone generator

**Item** Oxygen cylinder  **Quantity** 01 **Value** R$ 600,00 **Justification** Needed for ozone generation

**Prediction of compensation of expenses to the participants of the research**

There is no personal expenditures for the participant of the study at any point.

Either there is no financial compensation related to his / her participation.

**Right of indemnification**

In case of personal injury, directly caused for the procedures proposed in this study (proven causal link), the participant will have the right to medical treatment in the Institution, as well as the legally established indemnities.

8 Research monitoring

**Measures for the protection or minimization of any risks**

The procedures to which the participants of the research will be submitted (application of ozone or air in the knee) will be detailed, simulated and, only then, carried through.

All this will be carried through for the protection and minimization of any risks.

At all times, the participant of the research will be stimulated to make questionings about the study or the procedure to which he / she will be submitted.

**Measures of monitoring of data collection**

The quality of the Data Collection Forms will be conferred by comparison with the typed data. The conference will be carried through by two observers and the discords will be decided by a consensus meeting. The collected data will be stored in an electronic spread sheet (Microsoft Excel 2 for Windows 7, Microsoft Incorporation, Redmond, WA). Conference will be carried through by two observers and the discords will be decided by means of a consensus.

**Confidentiality measures**The confidentiality of the research participants will be maintained, and at no time or by any means there will be the possibility of public disclosure of results that will allow the identification of the research participants.

**Criteria for suspending or terminating the research**The survey will be suspended if the recruitment rate of the participants is less than 5 per week for 10 weeks. Research Ethics Committee will be notified if this occurs.

The research will be terminated if the parent electronic file (password protected in a hidden directory) is damaged or lost in its entirety, making it impossible to use the saved information. Research Ethics Committee will be notified if this occurs.

9 Risks and benefits  **The risks of the study are:**

The study presents only the risks inherent to knee puncture: joint infection, weakness and indisposition, and urticarial reactions due to suspension vehicle compounds. Other complications may be related to the local anesthetic used in the infiltrations. Accidental intravenous infiltration of lidocaine may cause severe reactions in the central nervous system or cardiovascular system. Side effects in the central nervous system include dizziness, visual changes, unintelligible pronunciation, and seizures. Cardiovascular reactions such as bradycardia, hypertension and cardio-respiratory collapse have been described. Immediate vasovagal reactions are common in patients with vasomotor instability (9).

The discomforts and risks due to intra articular use of ozone are immediate or delayed pain and fever, being considered of low complexity. The most frequent is an intense pain radiated for the leg or the muscle, with some minutes of duration, and that disappears spontaneously (5).

**Benefits of the study**

The benefits of the study are: improvement of hearing and vision, as well as disappearance of paresthesias in lower limbs and of the sensation of tiredness. Improvement in quality of life, daily and social activity, and improved agility and mood may also occur (5).

10 Properties of information and dissemination of research

The ownership of the results generated by this research is of its author. The results found at the end of the research will be published as an original article in an indexed journal, regardless if the research hypothesis is confirmed or not.

Copies of the research project and the partial and final reports of this research will be made available on the World Wide Web at the Cochrane Center in Brazil (http://www.centrocochranedobrasil.org.br) and delivered to the Internal Medicine and Therapeutics Discipline of Paulista School of Medicine - Federal University of Sao Paulo (EPM - UNIFESP) to obtain a Doctor degree in Sciences.

Bibliography

1. Schnitzer TJ. Osteoartrose (Doença óssea Degenerativa). In Bennett, JC; Plum, F. Cecil Tratado de Medicina Interna 20ª edição. Rio de Janeiro: Editora Guanabara Koogan; 1997. p. 1677-1681.
2. Leite RC. Terapias Bioxidativas 1ª edição. Curitiba: Corpo Mente Publicações; 1999. p. 18-43.
3. Leite RC. Ozônio 1ª edição. Curitiba: Corpo Mente Publicações; 1999. p. 5-17.
4. Al-Jazira AA; Mahmoodi SM. Painkilling effect of ozone-oxygen injection on spine and joint osteoarthritis. Saudi Medical Journal 2008; 29(4), 553-557. Dubai. United Arab Emirates.
5. Delgado Rifá Eraclio, Quesada Musa Juan Vicente. Ozonoterapia intraarticular en la enfermedad artrósica de rodilla. Rev Cubana Ortop Traumatol [revista en la Internet]. 2005 Jun [citado 2010 Jul 10] ; 19(1). Disponível em: http://scielo.sld.cu/scielo.php?script=sci_arttext&pid=S0864-215X2005000100005&lng=es.
6. Altman N. The Oxygen Prescription – The Miracle of Oxidative Therapies. Rochester: Healing Arts Press; 2007. p. 162 e 163.
7. Altman N. The Oxygen Prescription – The Miracle of Oxidative Therapies. Rochester: Healing Arts Press; 2007. p. 166.
8. Furtado R, Natour J. Infiltrações no aparelho locomotor – técnicas para realizaçao com e sem o auxílio de imagem. Porto Alegre: Artmed Editora S.A.; 2011.
9. Neustadt DH. Intra-articular injections for osteoarthritis of the knee. Cleveland Clinic Journal of Medicine. 2006.;73(10) 897-911.
10. Nagib H. Metodologia de Estudos em Ciências da Saúde – Como Planejar, Analisar e Apresentar um Trabalho Científico. São Paulo: Roca; 2.004.

Tables

**Table 1
Radiological criteria**

Classically the diagnosis of osteoarthritis has been based on radiological criteria since Kellgren and Lawrence, in 1957, published their work describing radiological changes of osteoarthritis. The following table shows the relevant aspects evidenced in this study for the radiological diagnosis of osteoarthritis.

| **01** | The formation of osteophytes on the joint margins or, in the case of the knee joint, on the tibial spines. |
| --- | --- |
| **02** | Periarticular ossicles; these were find chiefly in relation to the distal and proximal interphalangeal joints. |
| **03** | Narrowing of the joint cartilage associated with sclerosis of subchondral bone. |
| **04** | Small pseudocystic areas with sclerotic walls situated usually in the subchondral. |
| **05** | Altered shape of the bone ends, particularly in the head of femur. |

The combinations of these changes generate a scale of severity from 0 to 4 as the changes described above are absent (0), doubtful (1), minimal (2), moderate (3), and severe (4). However, some limitations evidenced in the study of Kellgren and Lawrence make difficult the evaluation of the joint space, since the knee xrays are made without load bearing.

**Kellgren JH, Lawrence JS. Radiological assessment of osteo arthrosis. Ann Rheum Dis. 1957;16(4):494-501.**

**Table 2
Diagnostic criteria of idiopathic knee osteoarthritis**

| **Clinical and laboratory** | **Clinical and radiographic** | **Clinical** |
| --- | --- | --- |
| Pain + at least 5 of 9 | Pain + at least 1 of 3 | Pain + at least 3 of 6 |
| Age > 50 years | Age > 50 years | Age > 50 years |
| Stiffness < 30minutes | Stiffness < 30minutes | Stiffness < 30minutes |
| Crepitus | Crepitus | Crepitus |
| Bony enlargement | + | Bony enlargement |
| Bony tenderness | Osteophytes | Bony tenderness |
| No palpable warmth | — | No palpable warmth |
| ESR < 40mm/hour | — | — |
| RF < 1:40 | — | — |
| SF OA | — | — |
| 92% sensitive | 91% sensitive | 95% sensitive |
| 75% specific | 86% specific | 69% specific |

* ESR = erythrocyte sedimentation rate (Westergren); RF = rheumatoid factor; SF OA = synovial fluid signs of OA (clear, viscous, or white blood cell count <2,W/mm3).

t Alternative for the clinical category would be 4 of 6, which is 84% sensitive and 89% specific.

**Altman R, Ash E, Bloch D, Bole G, Borenstein K, Brandt K, et al. Development of criteria for the classification and reporting os osteoarthritis. Classification of osteoarthritis of the knee. Diagnostic and therapeutic Criteria Committee of the American Rheumatism Association. Arthritis Rheum. 1986; 29(8):1039-49.**

Attachments

**Attachment 1**

**Model of data collection form**

**1. Identification**

| 0 | 1 | 2 | 3 | 4 | 5 | 6 | 7 | 8 | 9 |
| --- | --- | --- | --- | --- | --- | --- | --- | --- | --- |
| 0 | 1 | 2 | 3 | 4 | 5 | 6 | 7 | 8 | 9 |
| 0 | 1 | 2 | 3 | 4 | 5 | 6 | 7 | 8 | 9 |

**2. Researcher’s name**_______________________________________________________________________

**3. Date of completion** ___|___|_____

**4. Time of filling**  ___h___min

**5. Name of research participant**_______________________________________________________________________

**6. Sex** 1 Female 2 Male

**7. Date of birth**  ___|___|_____

**8. Profession**_______________________________________________________________________

**9. Height**  _______ m

**10. Weight** _______ kg

**11. Intensity of pain**_______________________________________________________________________

**12. Session of treatment**_______________________________________________________________________

**Attachment 2**

**Visual Analogic Scale (VAS)**


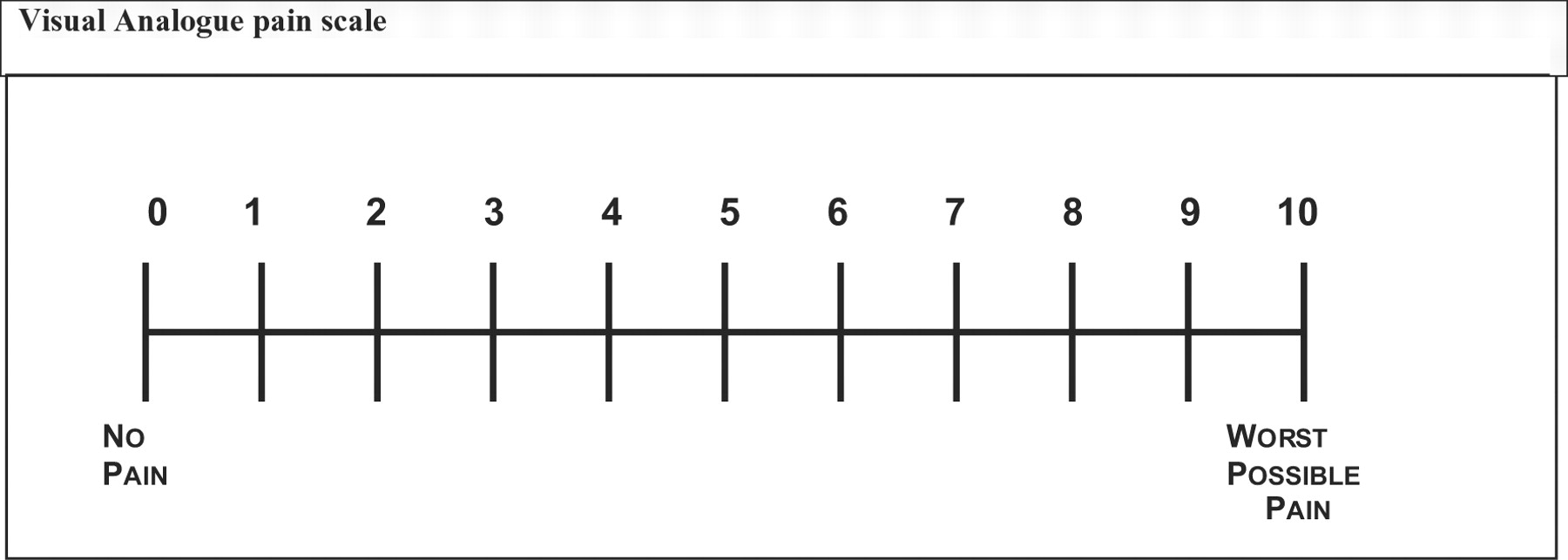


**Price DD. The validation of visual analogue scales as ratio scale measures for chronic and experimental pain. Pain. 1983; 17:45-56.**

**Attachment 3**

**Lequesne Functional Index**

**Pain or discomfort Points**

**During nocturnal bedrest**None 0 ⬜
Only on movement or in certain positions 1 ⬜
Without movement 2 ⬜

**Duration of morning stiffness or pain after getting up**None 0 ⬜
< 15 minutes 1 ⬜
>= 15 minutes 2 ⬜

**Remaining standing for 30 minutes increases pain**No 0 ⬜
Yes 1 ⬜

**Pain on walking**No 0 ⬜
Only after walking some distance 1 ⬜

Early after starting 2 ⬜

**Can you go up a standard flight of stairs?**No 0 ⬜
Yes 1 ⬜

**Maximum distance walked**Unlimited 0 ⬜
> 1 kilometer but limited 1 ⬜
About 1 kilometer (about 15 minutes) 2 ⬜
500 - 900 m (about 8 a 15 minutes) 3 ⬜
300 - 500 m 4 ⬜
100 - 300 m 5 ⬜
< 100 meters 6 ⬜

1 walking stick or crutch +1 ⬜
2 walking sticks or crutches +2 ⬜

**Difficulty of everyday life**Can you go up a standard flight of stairs? 0 - 2 _____
Can you go down a standard flight of stairs? 0 - 2 _____
Can you pick up an object from the floor? 0 - 2 _____
Can you walk on a irregular terrain? 0 - 2 _____

**Total**
0 No difficulty.
0,5 - 1,5 According to difficulty level.
2 Impossible.

**Adapted from: Lequesne MG. The algofunctional indices for hip and knee osteoarthritis. J Rheumatol. 1997; 24: 779-781.**

**Attachment 4
Timed Up and Go Test (TUG)**In TUG test, the patient is requested to stand up from a chair (height from seat= 45cm and from arms = 65cm), walk along 3 meters, come back and sit down again, while the time spent in making such work is timed. The proposition of such test is to evaluate the balance when sitting, transference from sitting position to standing one, stability when walking and changes when walking without using compensatory strategies. Independent individuals, without balance alterations, make the test within 10 seconds or fewer; with independence for basic transfers, they spent 20 seconds or fewer. Those individuals who need 30 seconds or more to finish the test are dependent in many daily life activities and to move, presenting greater risk of falling down.

**Podsiadlo D, Richardson S. The Timed “Up & Go”: a test of basic functional mobility for frail elderly persons. Journal of the American Geriatrics Society 1991; 39:142-48.Oliveira DLC, Goretti LC, Pereira LSM.**

**Attachment 5
SF-36 (Medical Outcomes Study Questionnaire Short Form 36 Health Survey)**

**1. In general, would you say your health is:**
1 Excellent
2 Very good
3 Good
4 Fair
5 Poor

**2. Compared to one year ago:**1 Much better now than one year ago
2 Somewhat better now than one year ago
3 About the same
4 Somewhat worse now than one year ago
5 Much worse now than one year ago

**3. The following items are about activities you might do during a typical day. Does your health now limit you in these activities? If so, how much (circle one number on each line)?**

**Vigorous activities, such as running, lifting heavy objects, participating in strenuous sports.**
1 Yes, limited a lot
2 Yes, limited a little
3 No, not limited at all

**Moderate activities, such as moving a table, pushing a vacuum cleaner, playing football, or sweeping.**1 Yes, limited a lot
2 Yes, limited a little
3 No, not limited at all

**Lifting or carrying groceries.**1 Yes, limited a lot
2 Yes, limited a little
3 No, not limited at all

**Climbing several flights of stairs.**1 Yes, limited a lot
2 Yes, limited a little
3 No, not limited at all

**Climbing one flight of stairs.**1 Yes, limited a lot
2 Yes, limited a little
3 No, not limited at all

**Bending, kneeling, or stooping.**

1 Yes, limited a lot
2 Yes, limited a little
3 No, not limited at all

**Walking more than a mile.**1 Yes, limited a lot
2 Yes, limited a little
3 No, not limited at all

**Walking several blocks.**
1 Yes, limited a lot
2 Yes, limited a little
3 No, not limited at all

**Walking one block.**1 Yes, limited a lot
2 Yes, limited a little
3 No, not limited at all

**Bathing or dressing yourself.**

1 Yes, limited a lot
2 Yes, limited a little
3 No, not limited at all

**4. During the past 4 weeks, have you had any of the following problems with your work or other regular daily activities as a result of your physical health (circle one number on each line)?**

**Cut down the amount of time you spent on work or other activities?**1 Yes
2 No

**Accomplished less than you would like?**1 Yes
2 No

**Were limited in the kind of work or other activities?**1 Yes
2 No

**Had difficulty performing the work or other activities (for example, it took extra effort)?**1 Yes
2 No

**5. During the past 4 weeks, have you had any of the following problems with your work or other regular daily activities as a result of any emotional problems (such as feeling depressed or anxious - circle one number on each line)?**

**Cut down the amount of time you spent on work or other activities?**1 Yes
2 No

**Accomplished less than you would like?**1 Yes
2 No

**Didn't do work or other activities as carefully as usual?**1 Yes
2 No

**6. During the past 4 weeks, to what extent has your physical health or emotional problems interfered with your normal social activities with family, friends, neighbors, or groups?**1 Not at all
2 Slightly
3 Moderately
4 Quite a bit
5 Extremely

**7. How much bodily pain have you had during the past 4 weeks?**1 None
2 Very mild
3 Mild
4 Moderate
5 Severe
6 Very severe

**8. During the past 4 weeks, how much did pain interfere with your normal work (including both work outside the home and housework)?**1 Not at all
2 A little bit
3 Moderately
4 Quite a bit
5 Extremely

**These questions are about how you feel and how were things like during the past 4 weeks. For each question, please give the one answer that comes closest to the way you have been feeling (circle one number on each line).**

**9. How much of the time during the past 4 weeks** **did you feel full of pep?**

1 All of the time
2 Most of the time
3 A good bit of the time
4 Some of the time
5 A little of the time
6 None of the time

**Have you been a very nervous person?**

1 All of the time
2 Most of the time
3 A good bit of the time
4 Some of the time
5 A little of the time
6 None of the time

**Have you felt so down in the dumps that nothing could cheer you up?**

1 All of the time
2 Most of the time
3 A good bit of the time
4 Some of the time
5 A little of the time
6 None of the time

**Have you felt calm and peaceful?**

1 All of the time
2 Most of the time
3 A good bit of the time
4 Some of the time
5 A little of the time
6 None of the time

**Did you have a lot of energy?**

1 All of the time
2 Most of the time
3 A good bit of the time
4 Some of the time
5 A little of the time
6 None of the time

**Have you felt downhearted and blue?**

1 All of the time
2 Most of the time
3 A good bit of the time
4 Some of the time
5 A little of the time
6 None of the time

**Did you feel worn out?**

1 All of the time
2 Most of the time
3 A good bit of the time
4 Some of the time
5 A little of the time
6 None of the time

**Have you been a happy person?**

1 All of the time
2 Most of the time
3 A good bit of the time
4 Some of the time
5 A little of the time
6 None of the time

**Did you feel tired?**

1 All of the time
2 Most of the time
3 A good bit of the time
4 Some of the time
5 A little of the time
6 None of the time

**10. During the past 4 weeks, how much of the time has your physical health or emotional problems interfered with your social activities (like visiting with friends, relatives, etc. - circle one number)?**

1 All of the time
2 Most of the time
3 A good bit of the time
4 Some of the time
5 A little of the time
6 None of the time

**11. How TRUE or FALSE is each of the following statements for you (circle one number on each line)?**

**I seem to get sick a little easier than other people.**
1 Definitely true
2 Mostly true
3 Don’t know
4 Mostly false
5 Definitely false

**I am as healthy as anybody I know.**1 Definitely true
2 Mostly true
3 Don’t know
4 Mostly false
5 Definitely false

**I expect my health to get worse.**1 Definitely true
2 Mostly true
3 Don’t know
4 Mostly false
5 Definitely false

**My health is excellent.**
1 Definitely true
2 Mostly true
3 Don’t know
4 Mostly false
5 Definitely false

**Ware JE, Sherbourne CD: The MOS 36 Item Short-Form Health Survey (SF-36). I. Conceptual framework and item selection. Med Care 30: 473-483, 1992.**

**Attachment 6
WOMAC**

**Section A: Pain intensity**

**What amount of knee pain do you experience?
1. Walking on flat surface.**□ None □ Mild □ Moderate
□ Severe □ Extreme

**2. Going up or down stairs.**□ None □ Mild □ Moderate
□ Severe □ Extreme

**3. At night while in bed.**□ None □ Mild □ Moderate
□ Severe □ Extreme

**4. Sitting or lying.**□ None □ Mild □ Moderate
□ Severe □ Extreme

**5. Straightening your knee fully.**□ None □ Mild □ Moderate
□ Severe □ Extreme

**Section B: Intensity of knee stiffness**

**1. How severe is your knee joint stiffness after first wakening in the morning?**□ None □ Mild □ Moderate
□ Severe □ Extreme

**2. How severe is your knee stiffness after sitting, lying or resting later in the day?**
□ None □ Mild □ Moderate
□ Severe □ Extreme

**Section C: Physical activity**

**Which is the degree of difficulty you experience due to your knee?
1. Descending stairs?**□ None □ Mild □ Moderate
□ Severe □ Extreme

**2. Ascending stairs?**□ None □ Mild □ Moderate
□ Severe □ Extreme

**3. Rising from sitting?**□ None □ Mild □ Moderate
□ Severe □ Extreme

**4. Standing?**□ None □ Mild □ Moderate
□ Severe □ Extreme

**5. Bending to floor?**□ None □ Mild □ Moderate
□ Severe □ Extreme

**6. Walking on flat surface?**□ None □ Mild □ Moderate
□ Severe □ Extreme

**7. Getting in/out of a car?**□ None □ Mild □ Moderate
□ Severe □ Extreme

**8. Going shopping?**□ None □ Mild □ Moderate
□ Severe □ Extreme

**9. Putting on socks/stockings?**□ None □ Mild □ Moderate
□ Severe □ Extreme

**10. Rising from bed?**□ None □ Mild □ Moderate
□ Severe □ Extreme

**11. Taking off socks/stockings?**□ None □ Mild □ Moderate
□ Severe □ Extreme

**12. Lying in bed?**
□ None □ Mild □ Moderate
□ Severe □ Extreme

**13. Getting in/out of bath?**□ None □ Mild □ Moderate
□ Severe □ Extreme

**14. Sitting?**□ None □ Mild □ Moderate
□ Severe □ Extreme

**15. Sitting or standing up from the toilet?**□ None □ Mild □ Moderate
□ Severe □ Extreme

**16. Doing heavy domestic duties?**□ None □ Mild □ Moderate
□ Severe □ Extreme

**17. Doing light domestic duties?**□ None □ Mild □ Moderate
□ Severe □ Extreme

**Bellamy N, Buchanan WW, Goldsmith CH, Campbell J, Stitt LW. Validation study of WOMAC: a health status instrument for measuring clinically important patient relevant outcomes to antirheumatic drug therapy in patients with osteoarthritis of the hip or knee. The Journal of Rheumatology. 1988;15(12):1833-184.**

**Attachment 7**

**Geriatric Pain Measure – GPM**

**Please answer each question:**

**1. Do you or would you have pain with vigorous activities such as running, lifting heavy objects or participating in strenuous sports?**
□ No □ Yes

**2. Do you or would you have pain with moderate activities such as moving a heavy table, pushing a vacuum cleaner, walking or playing soccer?**□ No □ Yes

**3. Do you or would you have pain with lifting or carrying groceries?**□ No □ Yes

**4. Do you or would you have pain with climbing more than one flight of stairs?**□ No □ Yes

**5. Do you or would you have pain with climbing only a few steps?**□ No □ Yes

**6. Do you or would you have pain walking more than one block?**□ No □ Yes

**7. Do you or would you have pain walking one block or less?**□ No □ Yes

**8. Do you or would you have pain with bathing or dressing?**□ No □ Yes

**9. Have you cut down the amount of time you spend on work or doing activities because of pain?**□ No □ Yes

**10. Does the work or activities you do require extra effort because of pain?**□ No □ Yes

**11. Have you been acomplishing less than you would like because of pain?**□ No □ Yes

**12. Does the work or activities you do require much effort because of pain?**□ No □ Yes

**13. Do you have trouble sleeping because of pain?**□ No □ Yes

**14. Does pain prevent you from attending religious activities?**□ No □ Yes

**15. Does pain prevent you from enjoying any other social or recreational activities (other than religious services)?**□ No □ Yes

**16. Does or would pain prevent you from traveling or using standard transportation?**□ No □ Yes

**17. Does pain makes you feel fatigued or tired?**□ No □ Yes

**18. Do you have to rely on family members or friends for help because of pain?**□ No □ Yes

**19. On a scale from zero to ten, with zero meaning no pain, with ten being the worst pain you can imagine, how severe is your pain today?**0 1 2 3 4 5 6 7 8 9 10

**20. In the last seven days, with zero meaning no pain, with ten being the worst pain you can imagine, how severe has your pain been on average?**0 1 2 3 4 5 6 7 8 9 10

**21. Do you have pain that never completely goes away?**□ No □ Yes

**22. Do you have pain every day?**□ No □ Yes

**23. Do you have pain several times a week?**□ No □ Yes

**24. Over the last seven days, has pain caused you to feel sad or depressed?**□ No □ Yes

**Scoring: Give one point for each yes response and add the numerical responses.**

**TOTAL SCORE (0 – 42): __________**

**Adjusted total score (Total Score X 238) (0 – 100): __________**

**Ferrell BA, Stein WM, Beck JC. The Geriatric Pain Measure: validity, reliability and factor analysis. J Am Geriatr Soc. 2000; 48(12):1669-1673.**

**Attachment 8
Model of Written Informed Consent Form**

COMPARISON BETWEEN INTRA ARTICULAR OZONE AND A PLACEBO IN THE TREATMENT OF KNEE OSTEOARTHRITIS

You are invited to participate in a research to obtain a better treatment of knee osteoarthritis in the elderly population. Your participation is very important for this research to be performed and for us to better treat knee osteoarthritis in the elderly, which leads to several limitations and disabilities. In this study, we will inject a gas called ozone or air in the knee of patients with osteoarthritis and aged between 60 and 85 years.

For this study, you are invited to a medical consultation, in which 6 questionnaires will be applied by the researcher doctor and, soon after your consent, it will be applied, in your diseased knee, ozone or air. You will receive 8 ozone or air injections. After the fourth and the eighth application, and 4 months after the applications are finished, you will respond again to the 6 questionnaires that will be applied by the researching physician.

The researcher is a physician at the Outpatient Clinic of the Institute of Geriatrics and Gerontology (IGG) of EPM-UNIFESP.

The discomfort that you will be able to have is related to the injection of the anesthetic (infection, allergic reaction and weakness, dizziness, vision and speech impairment) and ozone (fever and local pain).

You will not have any financial cost with the examinations and consultations carried out. There will also be no financial compensation for your participation. The results will be at your disposal at any time and the confidentiality of this information will be guaranteed. If you decide not to participate in the study, there will be no detriment to the continuity of your treatment at the institution.

At any stage of the study, you can clarify doubts with the medical team of the research (Dr. Carlos César Lopes de Jesus, Dr. Virgínia Fernandes Moça Trevisani and Dr. Fânia Cristina dos Santos, at Rua Francisco de Castro, 105 - tel. 5575-4848) or with the Research Ethics Committee (Rua Botucatu, 572 - 1st floor, set 14, tel 5571-1062).

Dr. Carlos César Lopes de Jesus is the main investigator of this research.

I, _______________________________________, declare to have been sufficiently informed about this research that seeks to improve the treatment of knee pain in the elderly population with osteoarthritis. I discussed with Dr. Carlos César Lopes de Jesus about my decision to participate, being clear the purpose of the study, its discomforts and risks, the guarantee of medical care, if necessary, confidentiality and permanent clarification. I know that my participation is free from financial costs or compensation and agree to participate voluntarily and may withdraw my consent at any time, without penalties in my attendance in this service.

Date: ____/____/____

Signature of patient or legal representative:
_______________________________________________________________________

Date: ____/____/____

Signature of the person responsible for reading the Term of Consent:
_______________________________________________________________________

**Attachment 9**

**Model of Term of Withdrawal of Written Consent Form**

The freedom to withdraw consent is guaranteed so that the patient stops participating in the study, without any type of harm to the individual.

I ______________________________________________ revoke the consent given on _______________ and affirm that I do not wish to continue in the study that was proposed to me, which I give as finalized on this date.

City: _________________________________________ Date: ____________________

Signature of researcher: ___________________________________________________

Signature of participant: __________________________________________________

Witness: _______________________________________________________________

**Attachment 10
Term of responsibility and commitment of the responsible researcher**

I, Carlos César Lopes de Jesus, researcher responsible for the project "Comparison between intra articular ozone and a placebo in the treatment of knee osteoarthritis", declare to be aware and that I will comply with the terms of Resolution 196 of 10/09/96 of the National Council of Ministry of Health. I also declare:

1. To make a commitment to ensure privacy and confidentiality of information;

2. To make the results of this research public whether they are favorable or not; and

3. To communicate the Research Ethics Committee of Paulista School of Medicine - Federal University of Sao Paulo (EPM - UNIFESP) about any changes in the research project, in semester reports or registered communication.

Sao Paulo, July 26th, 2010

_______________________________________________________________________
Carlos César Lopes de Jesus
Researcher

**Attachment 11
Statement of institutional conditions**

I, Álvaro Nagib Atallah, head of the Department of Internal Medicine and Evidence-Based Medicine of Paulista School of Medicine - Federal University of Sao Paulo (EPM - UNIFESP), declare to be aware of the terms of Resolution 196 of 10/09/96 and that the Department of Integral and Community Health has the resources and infrastructure to address possible problems resulting from the research project "Comparison between intra articular ozone and a placebo in the treatment of knee osteoarthritis."

Sao Paulo, July 26, 2010.

_______________________________________________________________________
Prof. Dr. Álvaro Nagib Atallah
Chief of Department of Internal Medicine and Evidence-Based Medicine
Paulista School of Medicine - Federal University of Sao Paulo (EPM - UNIFESP)

**Attachment 11
Researchers’ curriculum lattes**

**Adviser**Virgínia Fernandes Moça Trevisani, Master, PhD
*http://lattes.cnpq.br/9054730236021091*

**Second reader**Fânia Cristina dos Santos, Master, PhD
*http://lattes.cnpq.br/9874664960025710*

**Researcher**Carlos César Lopes de Jesus, Master
*http://lattes.cnpq.br/5070916128868023*

**Personal data
Name**Carlos César Lopes de Jesus

**Name in bibliographic citations**Lopes de Jesus, Carlos César

**Sex**Male

**Professional address**Escola Paulista de Medicina — Universidade Federal de São Paulo (EPM-UNIFESP)

Disciplina de Medicina Interna e Terapêutica
Rua Botucatu, 740 – 3º andar – Vila Clementino – São Paulo
Fone: +55 (11)5576-4203

**E-mail**caceloje@gmail.com

Consulted bibliography

Rother ET, Braga MER. **Como elaborar sua tese: estrutura e referências.** São Paulo: [s.n.]; 2005. 122 p.
